# Archaeal Sources of Intact Membrane Lipid Biomarkers in the Oxygen Deficient Zone of the Eastern Tropical South Pacific

**DOI:** 10.3389/fmicb.2019.00765

**Published:** 2019-04-11

**Authors:** Martina Sollai, Laura Villanueva, Ellen C. Hopmans, Richard G. Keil, Jaap S. Sinninghe Damsté

**Affiliations:** ^1^Royal Netherlands Institute for Sea Research (NIOZ), Department of Marine Microbiology and Biogeochemistry, Utrecht University, Den Burg, Netherlands; ^2^School of Oceanography, University of Washington, Seattle, WA, United States; ^3^Faculty of Geosciences, Utrecht University, Utrecht, Netherlands

**Keywords:** Thaumarchaeota, Euryarchaeota, DPANN, GDGT, archaeol, crenarchaeol

## Abstract

Archaea are ubiquitous in the modern ocean where they are involved in the carbon and nitrogen biogeochemical cycles. However, the majority of Archaea remain uncultured. Archaeal specific membrane intact polar lipids (IPLs) are biomarkers of the presence and abundance of living cells. They comprise archaeol and glycerol dibiphytanyl glycerol tetraethers (GDGTs) attached to various polar headgroups. However, little is known of the IPLs of uncultured marine Archaea, complicating their use as biomarkers. Here, we analyzed suspended particulate matter (SPM) obtained in high depth resolution from a coastal and open ocean site in the eastern tropical South Pacific (ETSP) oxygen deficient zone (ODZ) with the aim of determining possible biological sources of archaeal IPL by comparing their composition by Ultra High Pressure Liquid Chromatography coupled to high resolution mass spectrometry with the archaeal diversity by 16S rRNA gene amplicon sequencing and their abundance by quantitative PCR. Thaumarchaeotal Marine Group I (MGI) closely related to *Ca*. Nitrosopelagicus and *Nitrosopumilus* dominated the oxic surface and upper ODZ water together with Marine Euryarchaeota Group II (MGII). High relative abundance of hexose phosphohexose- (HPH) crenarchaeol, the specific biomarker for living Thaumarchaeota, and HPH-GDGT-0, dihexose- (DH) GDGT-3 and -4 were detected in these water masses. Within the ODZ, DPANN (Diapherotrites, Parvarchaeota, Aenigmarchaeota, Nanoarchaeota, and Nanohaloarchaea) of the Woesearchaeota DHVE-6 group and Marine Euryarchaeota Group III (MGIII) were present together with a higher proportion of archaeol-based IPLs, which were likely made by MGIII, since DPANN archaea are supposedly unable to synthesize their own IPLs and possibly have a symbiotic or parasitic partnership with MGIII. Finally, in deep suboxic/oxic waters a different MGI population occurred with HPH-GDGT-1, -2 and DH-GDGT-0 and -crenarchaeol, indicating that here MGI synthesize membranes with IPLs in a different relative abundance which could be attributed to the different detected population or to an environmental adaptation. Our study sheds light on the complex archaeal community of one of the most prominent ODZs and on the IPL biomarkers they potentially synthesize.

## Introduction

Archaea are numerous in the modern ocean (e.g., DeLong, 1992; Karner et al., 2001), where they constitute highly diverse communities (see Eme and Ford Doolittle, 2015 for a general review on the archaeal diversity) and participate in the biogeochemical cycles of elements (Offre et al., 2013). However, only a few members of marine Archaea have been isolated and their physiology remains therefore largely elusive.

Members of the phylum Thaumarchaeota (Brochier-Armanet et al., 2008), formerly known as Marine Group I (MGI) (DeLong, 1992; Fuhrman et al., 1992) have been successfully enriched and isolated (Könneke et al., 2005; Santoro et al., 2015). Marine Euryarchaeota Group II (MGII) and III (MGIII), whilst already detected by the pioneering studies of marine archaeal diversity (DeLong, 1992; Fuhrman et al., 1992), are yet to be isolated. The former group mostly includes motile photoheterotrophs living in the marine photic zone; other ecotypes, however, have been detected in deeper waters (Frigaard et al., 2006; Baker et al., 2013; Deschamps et al., 2014). MGIII have been mostly associated to oxic meso- and bathypelagic environments and are considered a rare component of deep-sea archaeal communities (Massana et al., 2000; López-García et al., 2001; Bano et al., 2004; Martin-Cuadrado et al., 2008; Haro-Moreno et al., 2017), albeit they were one of the most relevant lineages detected in the deep Arctic Ocean (Galand et al., 2009). A recent metagenomic analysis has found MGIII in epipelagic waters, containing genes of photolyases and rhodopsin that suggest a photoheterotrophic lifestyle (Haro-Moreno et al., 2017). Other uncultured planktonic archaeal lineages include members of the archaeal superphylum DPANN (Rinke et al., 2013; Castelle et al., 2015), which have recently also been described as important components of the euxinic waters of the Black Sea (Sollai et al., 2018).

In the last decade the abundance, environmental niche and involvement of yet uncultured Archaea into the marine biogeochemistry have started to be successfully investigated thanks to culture-independent techniques mostly based on genomic analyses (see Adam et al., 2017 for an updated review on the Archaea domain). These approaches, however, are subject to biases such as those regarding the primers used for amplification with PCR (see Teske and Sørensen, 2008; Pinto and Raskin, 2012; Marine et al., 2014; Eloe-Fadrosh et al., 2016 among others), and may be compensated by combining these techniques with other biogeochemical approaches (Hinrichs et al., 2000; Orphan et al., 2001; Biddle et al., 2006; Pitcher et al., 2011b). One successful example of this kind consists in coupling genomic and lipidomic techniques (Coolen et al., 2007; Wakeham et al., 2007, 2012; Pitcher et al., 2011b; Lipsewers et al., 2014; Besseling et al., 2018). Indeed, archaeal membrane lipids have proven to be promising taxonomic biomarkers and to be highly versatile in both present and past environments (Sinninghe Damsté et al., 2002), as they preserve better than DNA and RNA in soil and sedimentary records (Kuypers et al., 2001; Eglinton and Eglinton, 2008; Castañeda and Schouten, 2011). Archaeal membrane lipids are comprised of diphytanyl glycerol diether (archaeol) and/or glycerol dibiphytanyl glycerol tetraethers (GDGTs), which may contain 0–8 cyclopentane moieties. In general, GDGTs are widespread among the archaeal domain (Schouten et al., 2013). The only documented exception is crenarchaeol, the GDGT containing a cyclohexane moiety and four cyclopentanes (Sinninghe Damsté et al., 2002) which is considered exclusive to Thaumarchaeota (Sinninghe Damsté et al., 2012). In the lipid membranes of living archaea the core lipids (CLs) are attached by a glycerol-ether bond to one or two polar headgroups, which mainly include glyco- and phospholipids, forming structures known as intact polar lipids (IPLs; Sturt et al., 2004). Compared to CL, whose high preservation potential makes them suitable as biomarkers of both present and past archaeal communities (Schouten et al., 2013), IPLs have been suggested to be especially useful as biomarkers of the living archaea. Indeed, the death of cells leads to the hydrolysis of IPLs, which results in the prompt release of the polar headgroups. In particular, the phosphate-ester bond of the IPL phospho headgroup has been demonstrated to be more labile than the glycosidic ones that are found in the monohexose (MH) and dihexose (DH) IPL types, for instance (White et al., 1979; Harvey et al., 1986; Schouten et al., 2010). This feature makes the IPL hexose-phosphohexose (HPH)-crenarchaeol an excellent marker of living Thaumarchaeota (Pitcher et al., 2011a,b; Lengger et al., 2012). Studies of thaumarchaeotal cultures have demonstrated that HPH-crenarchaeol is indeed an important IPL (Schouten et al., 2008a; Pitcher et al., 2011a; Sinninghe Damsté et al., 2012) although its abundance seems to vary according to the growth phase (Elling et al., 2014).

The lack of cultures of marine archaea complicates the identification of specific biomarkers, similar to the HPH-crenarchaeol of the Thaumarchaeota group, for the various archaeal lineages. The use of DNA and biogeochemical tools to identify archaeal groups as sources of specific IPLs, directly in the environment, represents a valid alternative to the availability of isolates (Rossel et al., 2008; Pitcher et al., 2011b; Meador et al., 2015). Consequently, environments which sustain diverse archaeal communities are ideal for this type of investigation. Among these are those regions of the modern oceans hosting oxygen deficient zones (ODZs), areas where the concentration of oxygen is low enough that other electron acceptors are used for respiration (Horak et al., 2016). ODZs are characterized by multiple biogeochemical gradients which support complex archaeal communities taking part in alternative biogeochemical cycles such as the nitrogen, the carbon and in some settings the sulfur cycle (Canfield et al., 2010; Ulloa et al., 2012). Thaumarchaeota have been found to thrive and perform nitrification at the upper oxic–suboxic boundary of the main ODZs and other archaeal lineages are starting to be linked to the biogeochemistry of these regions (Lam and Kuypers, 2011). However, the presence and distribution of other archaeal groups in the ODZs are much less characterized, and so are the potential IPL biomarkers they synthesize *in situ*. Therefore, ODZs are interesting sites to study the presence of archaeal lineages and the IPLs they may produce.

Here, in order to expand the current knowledge on the distribution of marine archaea and their IPL biomarkers, we sampled a coastal and open ocean station of the eastern tropical South Pacific (ETSP) at relatively high depth resolution for suspended particulate matter (SPM). We characterized the archaeal diversity of the ETSP by 16S rRNA gene amplicon sequencing, and quantified the archaeal presence in the region by quantitative PCR. Archaeal IPL biomarkers were also investigated by using Ultra High Pressure Liquid Chromatography coupled to high resolution mass spectrometry (UHPLC-HRMS). In this way we were able to identify new archaeal IPL biomarkers and link them to specific archaeal groups.

## Materials and Methods

### Sampling and Physicochemical Determinations

**FIGURE 1:**
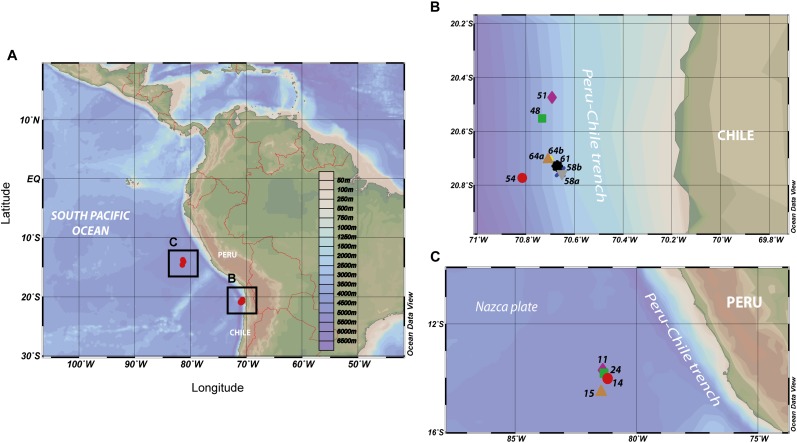
**(A)** Map of the eastern tropical South Pacific (ETSP) surveyed during the NBP13-0 cruise (June–July 2013) aboard of the *R/V Nathaniel B*. *Palmer*, including the location of the stations sampled for SPM by using multiple *in situ* pumps systems. Sampling locations **(B)** at the coastal station (i.e., 48; 51; 54; 58a; 58b; 61; 64a; and 64b) and **(C)** at the open ocean station (i.e., 11; 14; 15; and 24).

The ETSP water column was sampled during the NBP13-05 cruise aboard *R/V Nathaniel B*. *Palmer*, between 24th June and 27th July 2013 at coastal and open ocean sites (Figure 1). At each site a number of geographically nearby locations showing similar physicochemical features (based on the estimated potential density, oxygen and nutrient profiles) were surveyed as the ship tracked drifters placed in the ODZ. The Conductivity-Temperature-Density (CTD) equipment (SBE-911, Sea-Bird Electronics) recorded the physicochemical parameters of the water column. A SBE 43 electrochemical sensor mounted on the CTD rosette measured the dissolved-oxygen concentrations, which were calibrated against on-deck Winkler titrations. The electrochemical oxygen sensor SBE 43 has a detection limit of 1–2 μmol kg^-1^ (Tiano et al., 2014). Although recent evidence suggests that techniques such as Clark electrodes or Winkler titrations overestimate oxygen at the very lowest concentrations (Tiano et al., 2014), the O_2_ data here reported do not account for this discrepancy. Water samples for inorganic nutrient profiles were collected using a 24-bottle General Oceanics rosette sampler. Preferably, the CTD was cast shortly before or after the deployment of the McLane pumps (see below), otherwise data from another station, the closest in time and space to that of the deployed *in situ* pumps were used for the profiles. This means that the depths sampled for obtaining nutrient data do not always directly correspond to the depths at which the SPM was sampled with the *in situ* pumps.

The SPM was collected on 142 mm diameter pre-washed 0.7 μm pore size glass fiber (GF) filters (Whatman^®^), mounted in McLane WTS-LV *in situ* filtration systems. Two McLane pumps were deployed simultaneously at different depths, at each site. At the coastal site 15 depths from 22 to 1,000 m were sampled for IPL and gene analysis; at the open ocean site the samples were collected at nine discrete depths, comprised between 100 and 1,000 m. The volume of water filtered varied according to the depth and the material collected. Upon the recovery of the pumps the GF filters were removed, split in two halves, one of which was used for IPL and gene analysis and the other was archived, and both frozen at -80°C.

### DNA-Based Analysis

Suspended particulate matter filter halves were further subdivided into two quarter filters, which were used for genomic and for lipidomic analysis. DNA/RNA were extracted with the RNA PowerSoil^®^ Total Isolation Kit plus the DNA elution accessory (Mo Bio Laboratories, Carlsbad, CA, United States). The concentration of DNA was quantified by NanoDrop^TM^ spectrophotometer (Thermo Fisher Scientific, Waltham, MA, United States) and fluorometrically with Quant-iT^TM^ PicoGreen^®^ dsDNA Assay Kit (Life Technologies, Netherlands).

Amplification (primers: S-D-Arch-0159-a-S-15 and S-D-Bact-785-a-A-21; Klindworth et al., 2013) targeting bacterial and archaeal 16S rRNA gene reads and sequencing of the 16S rRNA gene amplicon was performed as described in Moore et al. (2015). The QIIME v1.9 software was employed to analyze the archaeal 16S rRNA gene amplicon sequences (Caporaso et al., 2010). The raw sequences were demultiplexed and subsequently quality-filtered with a minimum quality score of 25 length between 250 and 350, a maximum two errors in the barcode sequence were allowed. OTU picking step was performed with Usearch with a threshold of 0.97 (roughly corresponding to species-level OTUs). Taxonomy was assigned based on BLAST and the SILVA database version 123 (Altschul et al., 1990; Quast et al., 2013). The phylogenetic affiliation of selected 16S rRNA gene sequences was compared to release 123 of the Silva NR SSU Ref database^1^ (Quast et al., 2013) using the ARB software package (Ludwig et al., 2004). Sequences were added to the reference tree supplied by the Silva database using the ARB Parsimony tool.

Quantitative PCR (qPCR) was used to estimate archaeal 16S rRNA gene copies quantification, by using the primers Parch519F and Arc915R as previously described (Pitcher et al., 2011b). The qPCR reaction mixture (25 μl) contained 1 U of Pico Maxx high fidelity DNA polymerase (Stratagene, Agilent Technologies, Santa Clara, CA, United States) 2.5 μl of 10× Pico Maxx PCR buffer, 2.5 μl 2.5 mM of each dNTP, 0.5 μl BSA (20 mg/ml), 0.02 pmol/μl of primers, 10,000 times diluted SYBR Green^®^ (Invitrogen) (optimized concentration), 0.5 μl 50 mM of MgCl_2_ and ultrapure sterile water. All reactions were performed in iCycler iQ^TM^ 96-well plates (Bio-Rad, Hercules CA, United States). A gradient melting temperature assay was utilized to test the specificity of the reaction. The cycling conditions for the qPCR reaction consisted of 95°C, 4 min; 40–45 × [95°C, 30 s; 62°C, 40 s; 72°C, 30 s]; final extension 80°C, 25 s. The qPCR reactions were performed in triplicate with standard curves from 10^0^ to 10^7^ molecules per microliter. Efficiency of the qPCR assay was reported to be 98–100% and *R*^2^ = 0.996.

### Intact Polar Lipids Analysis

One fourth of each of the collected GF filters were used for lipidomic analysis. A modified Bligh-Dyer technique (Sturt et al., 2004) was used to extract IPLs, with some adjustments as reported by Sollai et al. (2018). Briefly, 1-*O*-hexadecyl-2-acetyl-sn-glycero-3-phosphocholine (PAF) internal standard was added to the extracts and dried under a stream of nitrogen. The IPL injection solvent (hexane:isopropanol:H_2_O 718:271:10 vol/vol/vol) was added to re-dissolved the dried extract and the resulting solution was filtered through a 0.45 μm, 4 mm-diameter True Regenerated Cellulose syringe filter (Grace Davison, Columbia, MD, United States). The analysis was performed as described by Sollai et al. (2018), by using an Ultimate 3000 RS UHPLC, equipped with thermostated auto-injector and column oven, coupled to a Q Exactive Orbitrap MS with Ion Max source with heated electrospray ionization (HESI) probe (Thermo Fisher Scientific, Waltham, MA, United States). Separation was achieved using an YMC-Triart Diol-HILIC column (250 mm × 2.0 mm, 1.9 μm particles, pore size 12 nm; YMC Co., Ltd., Kyoto, Japan) held at 30°C. The mass range for the analysis of the lipids was *m/z* 375 to 2,000 at a resolution 140,000. The chromatographic conditions and source settings were as described by Sollai et al. (2018), similarly the data dependent MS^2^ settings. The list of the IPLs targeted during the analysis, using a mass tolerance of 3 ppm, is also available in Sollai et al. (2018). Integration of the combined mass chromatogram (within a mass tolerance of 3 ppm) of the monoisotopic and first isotope peak of all relevant adducts formed was performed to determine the peak areas for each individual IPL. Depending on the type of IPL, protonated, ammoniated and/or sodiated adducts may form in different proportions. Possible matrix effects and variations in mass spectrometer performance were corrected when necessary by monitoring the response of the internal standard PAF. Reported peak areas were also corrected for these effects. The abundances of individual IPLs are here reported as response units per Liter of water (r.u. L^-1^), due to the lack of authentic standards for absolute quantitation. Although the MS response of individual IPLs may be different, the MS response of an individual IPL is stable when corrected for matrix effects and MS performance by the use of an internal standard as done here. This approach therefore allows for the comparison of absolute abundance (in terms of response units liter^-1^) between samples, but not the comparison of IPL relative abundance between the samples.

### Data Analysis

Pearson correlation analysis performed by using the R software package for statistical computing^2^ was applied to the data collected at both stations. Specifically the absolute abundance of specific archaeal groups, as calculated from the total archaeal 16S rRNA gene (copies L^-1^) estimated by qPCR (assuming one 16S rRNA gene copy number per genome) multiplied by the relative abundance of each archaeal group given by the 16S rRNA gene amplicon sequencing analysis detected in the ETSP SPM at different depths and the absolute abundance of the IPL classes (in terms of response units liter^-1^) as obtained with the UHPLC-HRMS analysis of the ETSP SPM at different depths were the data employed to build the correlation matrices. Three matrices were obtained. Type one (herein called archaea – IPL matrix) was built using the abundance of specific archaeal groups and the abundance of the IPLs detected as variables. Type two (herein called archaea – archaea matrix) was built using the abundance of specific archaeal groups as variables. Type three (herein called IPL – IPL matrix) used the abundance of the IPLs detected as variables. Data from 60 m depth at the coastal station were excluded from the analysis (see section “Discussion”). The correlation was expressed as coefficients (*r*-values) ranging from -1 to +1, where negative *r*-values indicate a negative linear correlation between the two variables, positive values indicate a positive linear correlation and 0 indicates no existing correlation.

## Results

**FIGURE 2:**
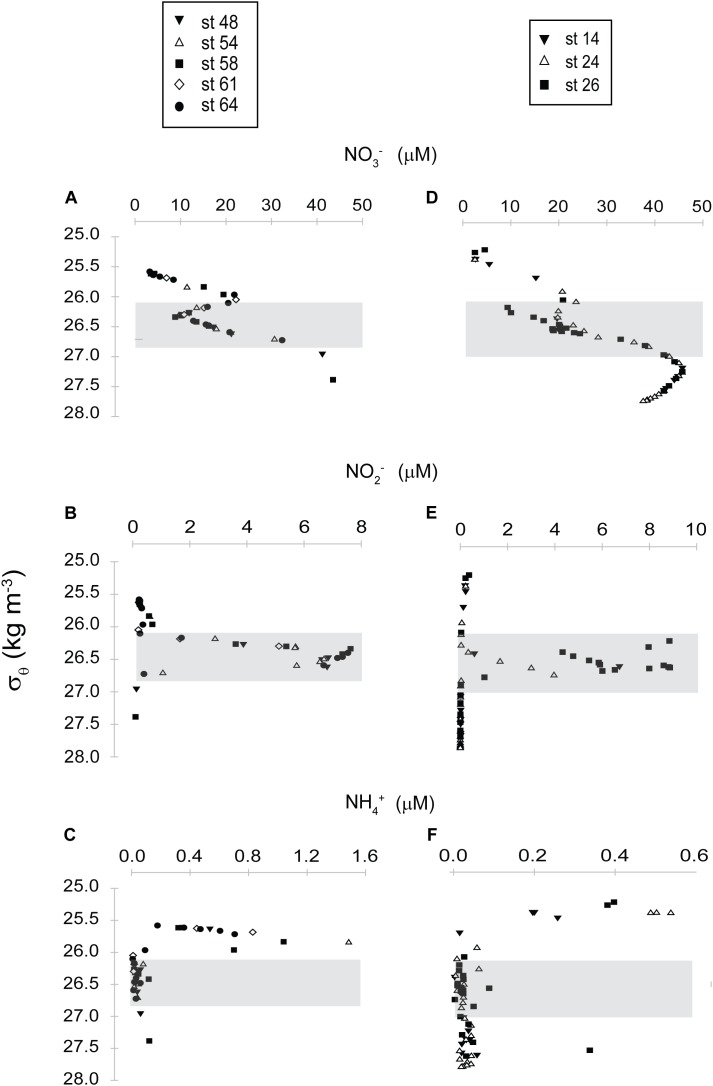
Profiles of nitrogen species detected (nitrate, NO_3_^-^; nitrite, NO_2_^-^; ammonium, NH_4_^+^) in the in the ETSP at the **(A–C)** coastal and **(D–F)** open ocean station.

The data obtained from casting geographically contiguous sites at both the study locations were compiled into two composite (i.e., coastal and offshore) stations (Figure 1). This was possible due to the large similarities of the physical and chemical profiles shared by the various sites from one location. The results are reported as tables or plots in which the potential density, σ_θ_ (kg m^-3^) corresponding to the depths sampled at the two stations are used (Supplementary Table S1 and Figure 1, 2).

### Physicochemical Parameters of the Sampling Sites

**FIGURE 3:**
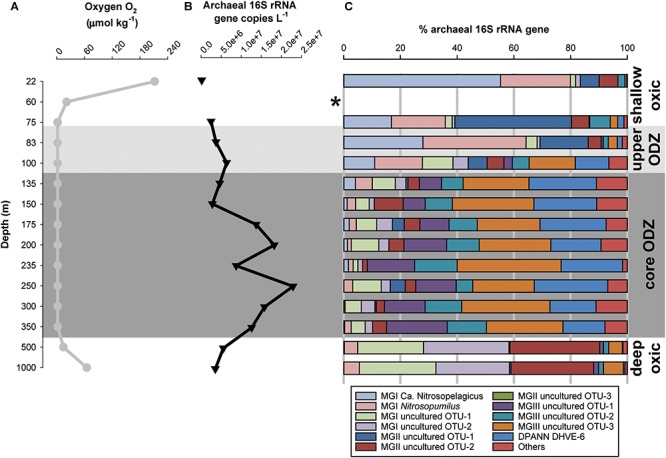
**(A)** Concentration profile of oxygen (O_2_, μmol kg^-1^), **(B)** absolute number of total archaeal 16S rRNA gene (copies L^-1^), and **(C)** percentage of the archaeal 16S rRNA gene reads of the archaeal groups detected across the ETSP water column at the coastal station. ^∗^At 60 m depth only 4 of the 8,127 recovered sequences were derived from archaea (0.1% of archaeal 16S rRNA gene reads), therefore we did not further consider this sample for analysis neither for discussion.

For the purpose of this study four distinct zones were defined in the coastal station, based on the O_2_ profile, and are used later in the text. These include the shallow oxic zone (22–75 m), the upper ODZ (75–135 m), the core ODZ (135–350 m), the deep oxic zone (350–1000 m; Figure 3). In detail, the water column of the coastal station was oxic for the first ∼70 m, with the O_2_ concentration reaching 212 μmol kg^-1^ at 22 m and 21.5 μmol kg^-1^ at 60 m, respectively. The oxycline was steep and at 75 m the water was suboxic (2.1 μmol kg^-1^ of O_2_). In the core ODZ (up to ∼350–400 m) the concentration of O_2_ remained close to 2 μmol kg^-1^. At ∼500 m the oxygen concentration increased to 14.6 μmol kg^-1^ (Supplementary Table S1 and Figure 3A). The concentrations of nitrate (NO_3_^-^), nitrite (NO_2_^-^) and ammonium (NH_4_^+^) were also determined. The NO_3_^-^ peaked in the upper oxycline (∼15–22 μmol L^-1^ at ∼40–60 m) and again in the deep oxycline (∼41–43 μmol L^-1^ at ∼500–1,000 m). The NO_3_^-^ concentration reached its lowest (∼9 μmol L^-1^) in the core ODZ, at ∼115 m (Supplementary Table S2 and Figure 2A). The NO_2_^-^ concentration was ∼0.7 μmol L^-1^ at ∼40–50 m and reached a peak of up to 7.5 μmol L^-1^ in the core ODZ, at ∼115–325 m (Supplementary Table S2 and Figure 2B). The NH_4_^+^ concentration peaked at ∼30 m (∼1.5 μmol L^-1^), it decreased to 0.1 μmol L^-1^ at ∼40 m and remained close to this value in the deeper water column (Supplementary Table S2 and Figure 2C).

**FIGURE 4:**
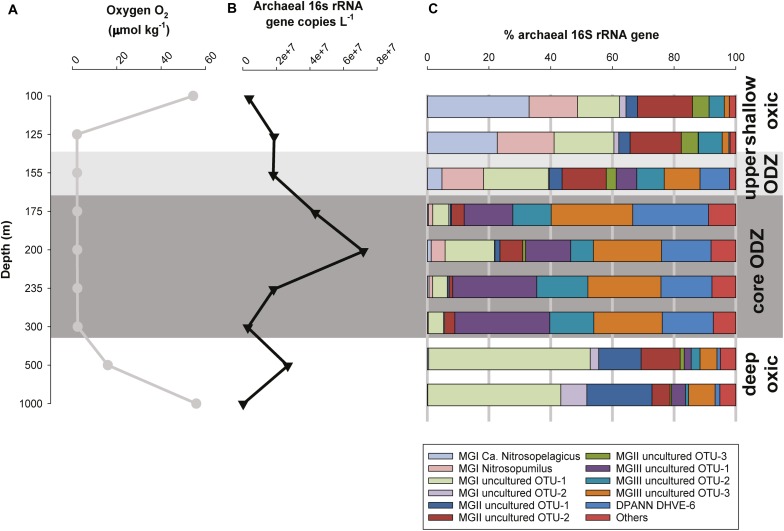
**(A)** Concentration profile of oxygen (O_2_, mmol kg^-1^), **(B)** absolute number of total archaeal 16S rRNA gene (copies L^-1^), and **(C)** percentage of the archaeal 16S rRNA gene reads of the archaeal groups detected across the ETSP water column at the open ocean station.

Similarly to the coastal station also in the open ocean site, four distinct zones were defined based on the O_2_ profile: the shallow oxic zone (100–125 m), the upper ODZ (125–175 m), the core ODZ (175–300 m), the deep oxic zone (300–1,000 m; Figure 4). At the open ocean station the water column was fully oxic (∼215 μmol kg^-1^) for the first 100 m and became suboxic in the following 25 m, with O_2_ decreasing to ∼2.1–5.5 μmol kg^-1^ at 125 m. Within the ODZ (∼125–300 m) the O_2_ was as low as ∼2–3 μmol kg^-1^, then it increased first to ∼11–16 μmol kg^-1^ at ∼500 m and to ∼56 μmol kg^-1^ at 1,000 m (Supplementary Table S1 and Figure 3A). The NO_3_^-^ concentration displayed a first maximum of ∼23.6 μmol L^-1^ at ∼100 m, it decreased to ∼20 μmol L^-1^ in the upper ODZ (∼150–160 m), and reached a second peak of ∼45.7 μmol L^-1^ at ∼700 m, in the lower oxycline (Supplementary Table S2 and Figure 2D). The NO_2_^-^ was barely detectable in the upper oxic water and at the oxycline (0.2 μmol L^-1^ at ∼50–60 m), but peaked (8.9 μmol L^-1^) within the core ODZ, at ∼120–270 m (Supplementary Table S2 and Figure 2E). The NH_4_^+^ concentration was at its maximum (0.5 μmol L^-1^) in the upper oxic water (∼50 m), then decreased to <0.1 μmol L^-1^ at ∼75–80 m, and did not increase again in the deeper water (Supplementary Table S2 and Figure 2F).

### Diversity and Abundance of Archaeal Groups

We investigated the archaeal community composition and abundance at both coastal and open ocean stations in the ETSP region. Partial 16S rRNA gene sequences were amplified and their sequence determined by amplicon sequencing of the DNA extracted from the SPM across the two water column vertical profiles (Table 1 and Figure 3, 4).

**Table 1:** Distribution of the archaeal 16S rRNA gene reads per depth, detected in the ETSP at the **(A)** coastal and **(B)** open ocean station.

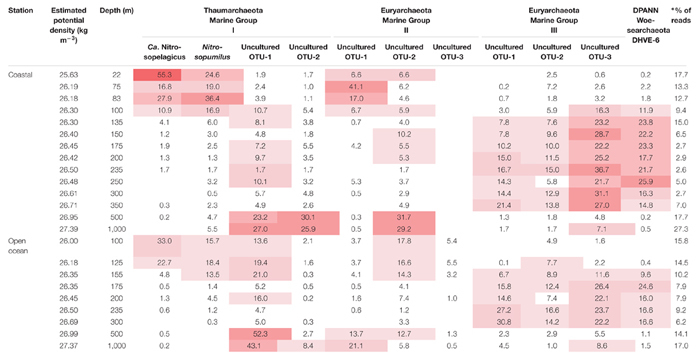

**^∗^Percentage of archaeal 16S rRNA gene reads of the total bacterial plus archaeal 16S rRNA gene reads detected in the analysis. Only archaeal groups with more than 3% of total archaeal reads are listed. The sample taken at 60 m depth only reported 0.1% archaeal 16S rRNA gene reads and archaeal 16S rRNA gene copy numbers under detection limit. For that reason, the sample is not reported in this table and was excluded from further analysis and discussion. Color coding: from dark red to white corresponds to 55–45%; 45–25%; 25–15%; 15–0% of archaeal 16S rRNA gene reads*.*

For both stations we quantified the total archaeal 16S rRNA gene with qPCR by using specific primers (Figure 3B, 4B and Supplementary Table S3). At the coastal station, the sample taken at 60 m depth only reported 0.1% archaeal 16S rRNA gene reads and archaeal 16S rRNA gene copy numbers under detection limit. For that reason, we excluded this sample from further analysis and discussion. At the coastal station the total archaeal 16S rRNA gene copy number was 2 × 10^5^ copies L^-1^ in shallow oxic water (22 m) and increased maximizing within the ODZ (175–350 m) with up to 2 × 10^7^ copies L^-1^. In deeper waters, it was ca. 3–5 × 10^6^ copies L^-1^ (Figure 3B and Supplementary Table S3A). At the open ocean station the total archaeal 16S rRNA gene copy number varied between 2 × 10^5^ to 7 × 10^7^ copies L^-1^. The maximum was located within the core ODZ (200 m) and a second maximum (3 × 10^7^ copies L^-1^) was detected at the lower oxycline at 500 m depth (Figure 4B and Supplementary Table S3B).

**FIGURE 5:**
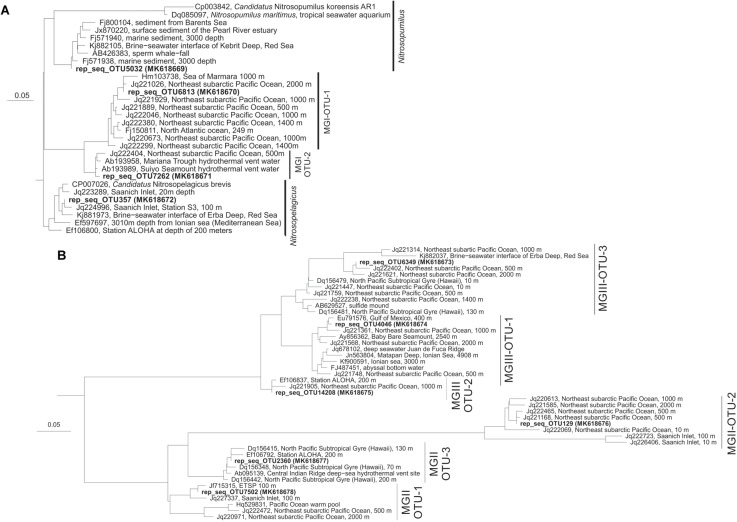
Phylogenetic trees showing the representative sequences (rep_seq, obtained by pick_rep_set.py in qiime of the OTUs picked by Usearch as specified in the material and methods. NCBI accession numbers between parentheses) present in the ETSP of the four OTUs of Thaumarchaeota **(A)**, and the three OTUs of MGII and MGIII archaea **(B)** in relation to closely related sequences. Scale bar indicates estimated sequence divergence (i.e., 5%).

Archaeal diversity was studied by 16S rRNA gene amplicon sequencing. At the coastal station, four major archaeal groups were detected, which could be related to various subgroups, i.e., the marine Thaumarchaeota (subdivided in operational taxonomic units, OTUs) closely related to *Candidatus* Nitrosopelagicus brevis and to *Nitrosopumilus maritimus*, and two other uncultured MGI OTUs: MGI OTU-1 and MGI OTU-2; Euryarchaeota MGII (as MGII OTU-1 and MGII OTU-2) and MGIII (as MGIII OTU-1, MGIII OTU-2, and MGIII OTU-3; see Figure 5 for details on the OTUs number and accession numbers), and uncultured DPANN Woesearchaeota Deep Hydrothermal Vent Group (DHVE)-6 (Figure 3C). At the open ocean station, the same archaeal taxa of the coastal station were detected with one more euryarchaeotal MGII subgroup (MGII OTU-3; Figure 5B). Other archaeal groups (less than 3% each) represented <10% of total archaeal reads (Figure 4C).

The archaeal groups showed a distinct distribution with depth. At the coastal station, Thaumarchaeota affiliated to *Ca*. Nitrosopelagicus and *Nitrosopumilus* dominated in oxic surface waters (at 22 m) with 55 and 25% of the total archaeal 16S rRNA gene reads, respectively. MGIII-affiliated OTUs were low (<2.5%) or undetected in the surface and subsurface water (ca. 22–83 m, Table 1 and Figure 3C). In the upper ODZ (75–83 m) MGII OTU-1 comprised 41 and 17% of reads, together with MGI archaea related to *Ca*. Nitrosopelagicus (11–28%) and *Nitrosopumilus* (19–36%). The distribution of these two groups of MGI archaea extended somewhat deeper in the water column; at 100 m MGI archaea (including MGI OTU-1 and MGI OTU-2) still comprised 44% of all archaeal reads. Within the core ODZ (∼135–350 m) the archaeal taxa that were prominent in the upper 100 m declined to <10% of archaeal reads in favor of other taxa. Specifically, euryarchaeotal MGIII OTU-3 increased gradually, reaching 42–63% at depths >150 m in the ODZ. In addition, archaea affiliated to the DPANN Woesearchaeota DHVE-6 group became increasingly abundant in the 135–350 m depth range, comprising up to 15–26% of total archaeal reads with the lowest relative abundance in the deeper part of the ODZ (Table 1 and Figure 3C). Below the ODZ, in the deep oxic water (500–1,000 m), the relative abundance of MGIII and DPANN DHVE-6-affiliated reads declined sharply, whereas MGI OTU-1 and MGI OTU-2 increased sharply to a summed relative abundance of >50%. In contrast to the surface oxic waters, MGI archaea related to *Ca*. Nitrosopelagicus and *Nitrosopumilus* were much lower in relative abundance in the deep oxic waters. Comparably, the relative abundance of MGII OTU-2 reads, but not of MGII OTU-1, also increased substantially to 29–32% (Table 1 and Figure 3C).

In general, the depth distribution of the archaeal taxa of the open ocean site was similar to that of the coastal site (cf. Table 1 and Figure 4C). The relative abundance of thaumarchaeotal *Ca*. Nitrosopelagicus and *Nitrosopumilus* reads in the oxic surface waters (100 m) was as high as 33 and 16%, respectively. In contrast to the coastal site, however, MGI OTU-1 was also relatively abundant (14%). MGI archaea remained the most prominent group of archaea in the upper ODZ (i.e., 40–62% of total archaeal reads at 125–155 m), but clear differences in the relative abundance of the various OTUs were observed. Specifically, *Ca*. Nitrosopelagicus-related archaea comprised 33% of reads at 100 m and decreased to <5% reads at 155 m, whereas MGI OTU-1 increased from 14 to 21% at the same depth range. The archaea related to *Nitrosopumilus* increased firstly to 18% and still represented 13% at 155 m. Another relevant group of archaea in the oxic surface waters was euryarchaeotal MGII, comprising 22–27% of total archaeal reads at 100–155 m. In contrast to the coastal station, MGII OTU-2 (14–18% of 16S rRNA gene reads) and not MGII OTU-1 was most prominent (Table 1 and Figure 4C). Like in the coastal station, the relative abundance of MGI (<6%) and MGII (<8%) archaea strongly decreased within the core ODZ (175–300 m), with the only exception being MGI OTU-1, whose reads reached 16% at 200 m. In contrast, the MGIII-affiliated OTUs and the DPANN group became prominent in the core ODZ, with relative abundances of individual OTUs reaching 30%. In the deep oxic waters (500–1,000 m) MGI OTU-1 was prominent again with 52 and 43% of reads. At these deeps other relevant archaeal taxa include MGII OTU-1 (14 and 21%) and MGII OTU-2 (13% at 500 m) and, to a minor extent, MGIII OTU-3 and MGI OTU-2 (8–9% at 1,000 m; Table 1 and Figure 4C).

### Archaeal Intact Polar Lipid Biomarkers

An UHPLC-HRMS method was set-up to detect 193 structurally different archaeal IPLs (see section “Materials and Methods” and Sollai et al., 2018). The distribution of individual IPLs versus depth (and corresponding estimated potential density, σ_θ_) was presented as the percentage of the relative abundance of each IPL (Supplementary Table S4) normalized for its total amount detected across the full depth profile (Tables 2, 3). Because no authentic standards were available for absolute quantitation of the individual IPLs (MS response factors of different IPLs might vary substantially), it was not possible to compare the relative abundances of the IPLs at one specific depth (see also section “Materials and Methods” for further explanation). 14 GDGT -IPLs and 4 archaeol-IPLs were detected in the ETSP in this study (Tables 2, 3). The IPLs detected included various head group types attached in different combinations to crenarchaeol, GDGT-0 to -4 and archaeol CLs. Specifically, they included monohexose (MH); dihexose (DH), which in this study stands for one DH moiety when attached to archaeol CL or for two MH moieties when attached to crenarchaeol or GDGT CLs; hexose phosphohexose (HPH), which stands for two headgroups, namely one MH and one phosphatidyl MH; phosphatidyl glycerol (PG), and phosphatidyl ethanolamine (PE).

**Table 2:** Depth distribution of the archaeal IPLs detected in the ETSP at the coastal station.

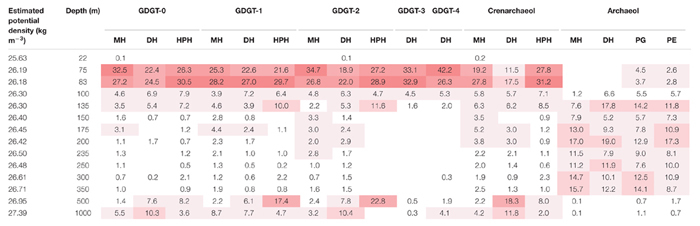

**These distributions are normalized on the summed abundance (response units per Liter, r.u. L^-*1*^) over all depths of each IPL detected. IPLs are listed according to the core lipid and the polar head groups attached to them. GDGT, glycerol dialkyl glycerol tetraether; MH, monohexose; DH, dihexose; HPH, hexose phospho-hexose; PG, phosphatidyl glycerol; PE, phosphatidyl ethanolamine. Color coding: from dark red to white corresponds to 42–32%; 32–22%; 22–12%; 12–0% of archaeal 16S rRNA gene reads. The table should be read in a vertical direction since the differences in MS response factors for different IPLs do not allow a direct comparison of the abundance of IPLs at one depth. IPL data from 60 m depth were not included neither discussed in the manuscript as the archaeal 16S rRNA abundance was under detection limit*.*

**Table 3:** Depth distribution of the archaeal IPLs detected in the ETSP at the open ocean station.

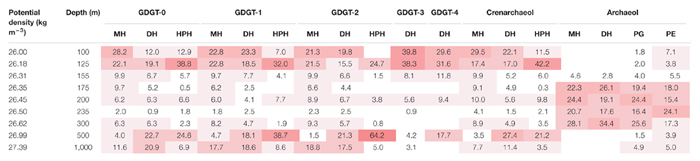

**These distributions are normalized on the summed abundance (response units per Liter, r.u. L^-*1*^) over all depths of each IPL detected. IPLs are listed according to the core lipid and the polar head groups attached to them. GDGT, glycerol dialkyl glycerol tetraether; MH, monohexose; DH, dihexose; HPH, hexose phospho-hexose; PG, phosphatidyl glycerol; PE, phosphatidyl ethanolamine. Color coding: from dark red to white corresponds to 64–42%; 42–32%; 32–22%; 22–12%; 12–0% of archaeal 16S rRNA gene reads. The table should be read in a vertical direction since the differences in MS response factors for different IPLs do not allow a direct comparison of the abundance of IPLs at one depth*.*

At the coastal station all IPLs with crenarchaeol and GDGT-0 to -4 as CLs showed similar depth profiles (Table 2). Specifically, these IPLs displayed their highest relative abundance in the upper ODZ (i.e., at 75–83 m) and gradually decreased within the core ODZ and deeper in the water column (see MH-crenarchaeol, DH-GDGT-3 and -4 for instance). The relative abundance of most HPH- and DH-GDGTs (except for DH-GDGT-3) sharply increased again at 500–1,000 m. At 1,000 m the relative abundance of the MH-GDGTs also increased. DH-crenarchaeol was an exception as it reached its highest relative abundance (18%) at 500 m depth. All archaeol-IPLs displayed a substantially different distribution being mostly detected in the core ODZ (Table 2). These IPLs were present with MH, DH, PG and PE attached as polar head groups. MH- and DH-archaeol were for the first time detected at 100 m depth, in the upper ODZ, while PG- and PE-archaeol were already detected in the upper oxycline, although at very low relative abundance (Table 2). Every archaeol-IPL type reached its own highest relative abundance at a different depth in the core ODZ: MH-archaeol generally increased in the deep ODZ, DH/PG/PE-archaeol, respectively, at 135, 200 and again at 300–350 m (Table 2).

At the open ocean station, the general distribution of the IPLs detected was rather similar to that of the coastal station (Table 3). Most of the crenarchaeol- and GDGT-IPLs had their highest relative abundance at the oxycline and the upper ODZ, which in this station corresponded to 100–125 m depth and then their abundance decreased within the core ODZ. As observed at the coastal station, for all HPH- and DH-GDGT-IPLs, but not for MH-GDGT-IPLs, the relative abundances increased sharply again at 500 m (Table 3), whilst the abundance of MH-GDGTs showed an increase in the deepest water layer studied (i.e., 1,000 m). In contrast with the coastal station, for the open ocean station the maximum relative abundance often occurred in the deep oxycline (below 300 m). This was the case for DH-crenarchaeol, DH-GDGT-0 and -2, HPH-GDGT-1 and, especially, -2. As for the coastal station also for the open ocean station, the archaeol-based IPLs were prominent only in the core ODZ (Table 3). In particular, MH- and DH-archaeol were only detected at 155–300 m and showed their highest relative abundance (28 and 34%, respectively) in the deep ODZ, at 300 m. PG- and PE-archaeol, although present at all depths, also had their highest relative abundance within the ODZ (175–300 m), specifically at 300 m for PG-archaeol (26%) and at 235 m for PE-archaeol (24%; Table 3).

### Statistical Analysis

**FIGURE 6:**
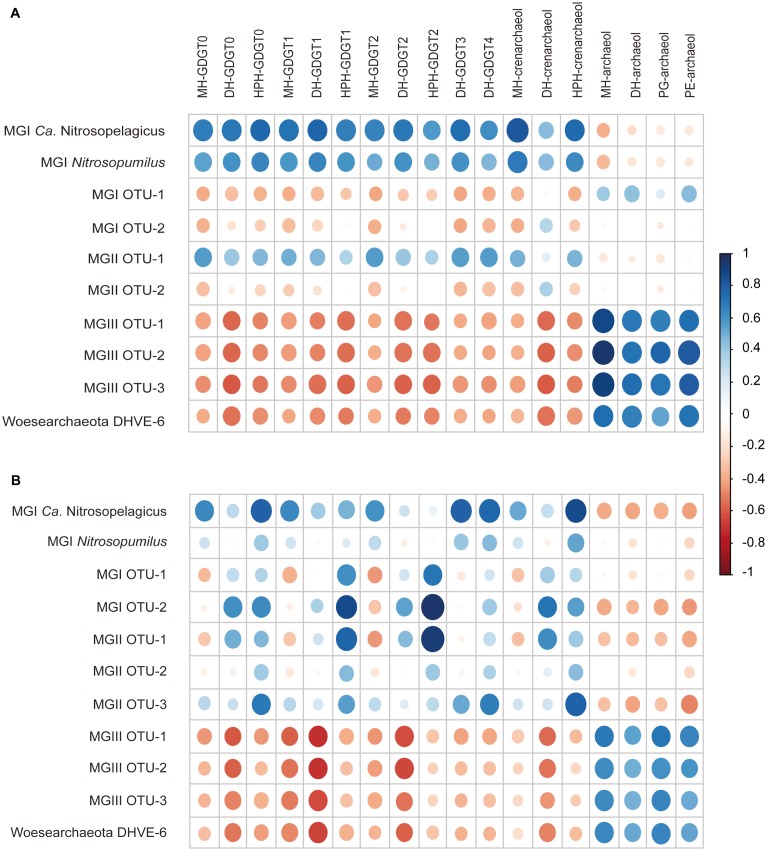
Dot plot of the correlation matrix obtained by applying a Pearson analysis to the total archaeal 16S rRNA gene reads (copies L^-1^) of the archaeal groups and to the abundances of the archaeal IPLs (response units per Liter; r.u. L^-1^) detected at the **(A)** coastal and **(B)** open ocean stations of the ETSP. Dark blue corresponds to +1 *r*-values, indicating a strong positive linear correlation between archaeal groups and IPLs; white corresponds to 0 *r*-values, indicating that no correlation exists; dark red corresponds to –1 *r*-values, indicating a strong negative linear correlation.

The archaea – IPL matrix (Figure 6 and Supplementary Table S5) aimed to corroborate or dismiss the tentative assignment of specific IPLs to specific archaeal groups. Both coastal (Figure 6A and Supplementary Table S5A) and open ocean archaea – IPL matrices (Figure 6B and Supplementary Table S5B) revealed clear patterns of positive and negative correlations between various archaeal groups and archaeal IPLs in the water column of the ETSP, suggesting potential relationships between the variables that will be discuss later in detail. The archaea – archaea matrix identified possible biases in the correlations between archaeal groups and IPLs (Supplementary Figure S1 and Supplementary Table S6). The IPL – IPL matrix revealed potentially similar sources of IPLs (Supplementary Figure S2 and Supplementary Table S7).

## Discussion

The biogeochemistry of the ETSP ODZ has been extensively studied with an emphasis on the nitrogen cycle (see Lam et al., 2009; Canfield et al., 2010; Ulloa et al., 2012 among others). Thaumarchaeota have attracted most of the attention among archaea, because they perform the oxidation of ammonium to nitrite in a broad range of O_2_ regimes (Coolen et al., 2007; Lam et al., 2007, 2009; Beman et al., 2008; Molina et al., 2010; Pitcher et al., 2011b; Bristow et al., 2016). In our study, we observed a higher archaeal diversity in the ESTP water column than previously reported (Quiñones et al., 2009; Belmar et al., 2011; Stewart et al., 2011), and a clear niche occupancy of archaeal groups (Figure 3, 4). Archaea were more abundant in the core ODZ than in the shallow or deep oxic zones (Figure 3B, 4B). This was particularly evident in the coastal station where the archaeal 16S rRNA gene abundance was at least one order of magnitude higher in the core ODZ compare to the rest of the water column (cf. Figure 3B, 4B). This distribution overall suggests that the archaeal groups detected within the ETSP ODZ might have a relevant role in the biogeochemistry of these waters. In our study, we applied a combined IPL and gene-based approach which has already proven successful in other locations with similar features to the ETSP, including the Arabian Sea (Pitcher et al., 2011b; Schouten et al., 2012; Besseling et al., 2018) and the Black Sea (Sollai et al., 2018). However, the use of UHPLC-HRMS greatly improves the IPL approach as it makes possible to reveal a broader IPL diversity (Wörmer et al., 2013; Zhu et al., 2013; Xie et al., 2014; Liu et al., 2016) and its taxonomic potential, normally a substantial limitation of the approach, thus improving the association of these lipids with their biological sources (Besseling et al., 2018; Lipsewers et al., 2018). Here, we also apply a statistical analysis to tentatively assign the detected IPLs to specific archaeal lineages.

### A Diverse Thaumarchaeotal Population Resides in the Shallow Oxic Waters, the Upper ODZ, and in the Deep Oxic Waters of the ETSP

Thaumarchaeota formed a prominent component of the archaeal population from the shallow oxic waters down to the upper ODZ at both stations: *Ca*. Nitrosopelagicus was dominant over *Nitrosopumilus* and MGI OTU-1 at the uppermost depth and MGI OTU-2 (Figure 5A) was only detected in low relative abundance (Table 1 and Figure 3C, 4C). At the open ocean station, where sampling only started at 100 m, we suspect the same distribution also at shallower depths. MGI *Ca*. Nitrosopelagicus, although genetically closely related to *Nitrosopumilus*, is expected to be better adapted to the pelagic environment (Santoro et al., 2015). Its higher relative abundance at the shallower depths might be due to a better response to photo-inhibition stress or to a more efficient uptake of ammonium (Merbt et al., 2012; Smith et al., 2014; Santoro et al., 2015). At both stations *Ca*. Nitrosopelagicus tended to decrease in relative abundance toward the upper ODZ, confirming the preference of this group for a shallow oxic water niche (Table 1 and Figure 3C, 4C). On the contrary, at the coastal station *Nitrosopumilus* maximized in the upper ODZ at 83 m (Table 1 and Figure 3C). At the open ocean station *Nitrosopumilus* relative abundance remained stable down to the oxycline (18% at 125 m) and in the upper ODZ (13% at 155 m; Figure 4C). The high affinity of *Nitrosopumilus* for NH_4_^+^ (Martens-Habbena et al., 2009) and its proven adaptability to micro to nanomolar O_2_ concentrations (Bristow et al., 2016) are probably among the reasons why members of this group occupied that specific niche at the two stations. At the open ocean station, the relative abundance of sequences affiliated to the MGI OTU-1 increased from the shallow oxic waters (14% at 100 m) to the oxycline and then remained stable in the upper ODZ (ca. 20% at 125–155 m; Table 1 and Figure 4C). A higher relative abundance of Thaumarchaeota in the oxycline/upper ETSP ODZ has been previously reported by archaeal *amo*A gene copy numbers, CARD-FISH and protein-coding transcript counts attributed to Thaumarchaeota (Lam et al., 2009; Molina et al., 2010; Belmar et al., 2011; Stewart et al., 2011). Additionally, the tendency of Thaumarchaeota niche to occur at the oxycline/upper ODZ has been described as a recurring feature in the other main ODZs such as the eastern tropical North Pacific (ETNP) and the Arabian Sea ODZs (Beman et al., 2008, 2012; Pitcher et al., 2011b), and anoxic basins such as the Black Sea, the Cariaco Basin, and the Gotland Basin of the Baltic Sea (Coolen et al., 2007; Wakeham et al., 2012; Berg et al., 2014).

Within the core ODZ, Thaumarchaeota became a minor component of the archaeal community at both stations, only making up <20% of the 16S rRNA gene reads (Table 1 and Figure 3C, 4C). Specifically, whereas MGI OTU-1 maintained some relevance at both stations, *Ca*. Nitrosopelagicus and *Nitrosopumilus* decreased substantially within the archaeal population (Table 1 and Figure 3C, 4C). This pattern is consistent with previous studies (Belmar et al., 2011; Stewart et al., 2011) and suggests that the Thaumarchaeota were not well adapted to the core ODZ, likely because of the lack of oxygen. However, the thaumarchaeotal groups remained detectable throughout the ODZ (Figure 3, 4). This might be simply due to the preservation of DNA which once produced in the surface oxic waters was transported/sink in the ODZ where was maintained as fossil, favored by anoxia, confirming the founding of a recent study from the Arabian Sea in which fossil 16S rRNA and *amo*A gene copies were found in anoxic sediments (Besseling et al., 2018). Interestingly, in a previous study Peng et al. (2013) reported a higher number of *amo*A gene copies in the core ETSP ODZ off the Peruvian coastline (i.e., at 260 m) compared to the oxic surface (i.e., at 20 m) and suggested that within the ODZ the thaumarchaeotal cells were dormant or able to perform an alternative energetic metabolism to aerobic ammonia oxidation to nitrite. However, the occurrence of periodic aerobic metabolisms including ammonia oxidation, likely fueled by episodic intrusions of O_2_ within the ODZ (Ulloa et al., 2012; Bristow et al., 2016), cannot be ruled out.

At both stations, Thaumarchaeota regained relevance below the ODZ (below 350 m at the coastal station and below 300 m at the open ocean station) dominating the archaeal community at those depths (Table 1 and Figure 3C, 4C). In particular, at the coastal station, *Nitrosopumilus* was present low abundances, whereas the two uncultured MGI OTUs (Figure 5A) were particularly abundant (Table 1 and Figure 3C, 4C). The MGI OTU-1 and OTU-2 detected in this study are closely related to sequences previously detected in the upper oxycline, within the ODZ and in the deep oxycline of the Northeast Subarctic Pacific Ocean oxygen minimum zone (NESAP OMZ; Figure 5A). At the time of sampling, the core of the NESAP ODZ was characterized by O_2_ concentrations of 8.6–15 μmol kg^-1^ and in some cases even reached 60 μmol kg^-1^ (Freeland et al., 1997; Whitney et al., 2007). We found similar oxygen concentrations in the upper oxycline and deep oxic waters of the ETSP (Figure 3A, 4A), which finding supports the idea that these sequences represent Thaumarchaeota adapted to waters with oxygen levels in the range of approximately 9–60 μmol kg^-1^. In the deep oxic waters MGI OTU-1 and MGI OTU-2 had similar abundance at the coastal station, whereas at the open ocean the former OTU was more abundant. However, in general these two OTUs had a much higher relative abundance in the deep oxic water in comparison with the archaea closely related to *Nitrosopumilus* and *Ca*. Nitrosopelagicus. This specific ‘deep water’ niche occupancy of the MGI has been previously observed for many locations including the ETSP, the ETNP and the Arabian Sea ODZs among others, with the presence of a shallow cluster (i.e., cluster A) genetically different from the deep water cluster (i.e., cluster B) (Francis et al., 2005; Hallam et al., 2006; Mincer et al., 2007; Beman et al., 2008; Santoro et al., 2010; Belmar et al., 2011; Hu et al., 2011; Villanueva et al., 2015). Previous studies have suggested ammonia availability as a driver of the diversification of Thaumarchaeota in the water column (Sintes et al., 2016 among others). However, the concentrations of ammonia reported here throughout the water column (Figure 2C,F and Supplementary Table S2) are low and similar, which suggests that alternative factors other than ammonia and oxygen levels may play a role in this differentiation.

Based on their relevant presence in the shallow oxic zone of the ETSP, at both stations, MGI *Nitrosopumilus*, MGI *Ca*. Nitrosopelagicus and the MGI OTU-1 are likely to be the predominant source of the IPLs that were found in these waters down to the upper ODZ (Table 1 and Figure 3C, 4C). This hypothesis would be in good agreement with culture studies of the lipidome of *Nitrosopumilus* which revealed that MH/DH/HPH-GDGT-0–4 and -crenarchaeol IPLs comprise its major membrane lipids (Schouten et al., 2008a; Elling et al., 2014, 2017). HPH-IPLs are believed to be most prone to degradation and are therefore believed to be the best ‘life markers’ for Thaumarchaeota (Pitcher et al., 2011a; Schouten et al., 2012). Indeed, Elling et al. (2014) showed that HPH-crenarchaeol is mostly synthesized when cells are actively growing (Elling et al., 2014). Here we report that in the ETSP HPH-crenarchaeol was especially abundant at the boundary between the upper oxycline and the upper ODZ (75–83 m at the coastal station, and at 125 m at the open ocean station; Tables 2, 3), which was also where MGI *Nitrosopumilus* and MGI OTU-1 were most abundant (Table 1 and Figure 3C, 4C). This suggests that these were living populations, and confirms a preferred niche of *Nitrosopumilus* and, possibly, MGI OTU-1 for suboxic conditions. The hypothesis of the thaumarchaeotal origin of the archaeal IPLs found in the oxic surface waters was also confirmed by our archaea – IPLs correlation matrix (Figure 6). Indeed, this revealed that at the coastal station both *Ca*. Nitrosopelagicus and *Nitrosopumilus* had the highest correlation with the MH/DH/HPH-GDGT-0–4 and -crenarchaeol IPLs (*r*-value > 0.60 and > 0.50; Figure 6A and Supplementary Table S5A). At the open ocean site instead, these positive correlations were confirmed for *Ca*. Nitrosopelagicus but somehow were less evident for *Nitrosopumilus* (Figure 6B and Supplementary Table S5B). Together, our findings indicate that both *Nitrosopumilus* and *Ca*. Nitrosopelagicus were actively growing and suggest that they were the main contributors to the IPL inventory in the shallow oxic and suboxic waters of the ETSP.

These IPLs (i.e., GDGT-0–4 and crenarchaeol with MH/DH/HPH-headgroups) had been previously reported in other similar locations, including the Arabian Sea, where MH/DH/HPH-GDGT-0–3 and -crenarchaeol peaked in the upper ODZ (Schouten et al., 2012), and the ETNP, where MH/DH-GDGT-0, -2 and -crenarchaeol were also especially abundant in the upper ODZ (Xie et al., 2014). In contrast, in the Cariaco Basin MH-GDGT-0–3 and -crenarchaeol were detected in the oxycline, whereas DH-GDGT-0–3 and -crenarchaeol were found in the deeper suboxic waters (Wakeham et al., 2012). In the Black Sea, albeit with different individual distributions, MH/DH/HPH-GDGT-0–4 and -crenarchaeol were also present in the oxic surface water and remained prominent down to the upper suboxic zone (Schubotz et al., 2009; Sollai et al., 2018).

The PE/PG-archaeol IPLs were also detected in the shallow oxic zone of the ETSP, although here their relative abundance was low compared to the upper and especially the core ODZ where they became much more abundant (below 135 m at the coastal station and below 175 m at the open ocean station; Tables 2, 3). All archaeal lineages thriving in the shallow oxic zone showed a negative correlation with these IPLs, with the exception of MGI OTU-1 at the coastal station (*r*-values ca. 0.20–0.46; Figure 6 and Supplementary Table S5) suggesting that the thaumarchaeotal population was not strongly contributing to the synthesis of the PE/PG-archaeol IPLs in the shallow oxic ETSP waters.

After a strong decline in the core of the ODZ, the GDGT-0–4 and crenarchaeol IPLs became prominent again in the deep oxic waters (Tables 2, 3). However, here the distribution of the archaeal IPLs did not revert completely to the one observed in the shallow oxic waters; the HPH-GDGT-1, -2, DH-GDGT-0 and -crenarchaeol became especially abundant resulting in a distinct distribution of archaeal IPLs (Tables 2, 3). At both stations, these four IPLs showed a strong positive correlation with each other in the IPL – IPL matrix, suggesting a common thaumarchaeotal origin (Supplementary Figure S2 and Supplementary Table S7). At the open ocean station the HPH-GDGT-2 was especially prominent with a distinct maximum in its relative abundance at 500 m (Table 3) that was almost three times higher than in the shallow oxic waters. Earlier studies from other oceanic regions have shown that the fractional abundance of GDGT-2 relative to other core GDGTs increases with increasing water depth and that this trend is more pronounced for IPL-GDGTs as for CL-GDGTs (Taylor et al., 2013; Hernández-Sánchez et al., 2014; Kim et al., 2015, 2016; Villanueva et al., 2015). Since at these depths the GDGT-2 was mostly found attached to the HPH headgroup (the most labile form for an archaeal IPLs) we infer that GDGT-2 was likely actively produced in the deep oxic waters of the ETSP. At the open ocean site the abundance of HPH-GDGT-2 had a highly positive correlation with the copy number of MGI OTU-2, and to a slightly lesser degree, of MGI OTU-1 (Figure 6B and Supplementary Table S5), the two Thaumarchaeota thriving in the deep oxic zone. Positive correlations were also observed between these two thaumarchaeotal groups and DH- and HPH-GDGT-0, -GDGT-1, and -crenarchaeol. At the coastal site, these correlations were not as evident, although most of the IPLs named above showed a clear sub-maximum at 500 m (Table 2). This might be due to the fact that all the four different groups of Thaumarchaeota detected, produce the same type of IPLs but in a different distribution. Overall these results suggest that the IPLs found in the deep oxic zone of the ETSP were synthesized by the thaumarchaeotal groups detected at those depths (Figure 6 and Supplementary Table S5).

### Marine Euryarchaeota Group II (MGII) Co-exists With the Shallow and Deep Thaumarchaeotal Populations

At the coastal station the MGII population was formed by two uncultured OTUs, whose abundance increased with depth. At 75 m MGII had become the second most abundant archaeal group after the Thaumarchaeota accounting for 47% of the total archaeal reads, and MGII OTU-1 was clearly dominant over MGII OTU-2 (41% compared to 6%). At the open ocean site, the MGII shallow population included a third OTU (i.e., MGII OTU-3) that was not found at the coastal station (cf. Figure 3C). At this station MGII OTU-2 was clearly the most abundant MGII OTU (14–18% compared to ca. 4% of MGII OTU-1 and ca. 5% of MGII OTU-3; Table 1 and Figure 4C). The distribution displayed by MGII in the shallow oxic waters and upper ODZ of the ETSP at both stations agrees with precedent reports in which the group was found as particularly prominent from the shallow oxic waters (ca. 0–50 m) down to the core ODZ (to ca. 200 m), although to a lower extent (Quiñones et al., 2009; Belmar et al., 2011). This suggests that, especially at the open ocean station where the sampling started from 100 m depth, the group might have been present also in shallower oxic waters (Hugoni et al., 2013). In fact, MGII are believed to have a heterotrophic lifestyle and a putative proteorhodopsin gene, whose expressed protein, powered by light, would allow the cells to move toward preferential food sources, has been found in the genomes of MGII detected in the photic zones in the North Pacific Ocean (Frigaard et al., 2006; Iverson et al., 2012). Their abundance in the shallow ETSP is also in line with findings from other locations worldwide (Massana et al., 2000; Galand et al., 2009; Iverson et al., 2012; Podlaska et al., 2012).

The sequences affiliated to MGII OTU-1 and MGII OTU-2 detected by this study were closely related to those previously detected in the ETSP and in the Saanich inlet at 100 m depth, and to sequences detected in the NESAP oxygen minimum zone (OMZ) at various depths (Figure 5B). Finally, sequences affiliated to the MGII OTU-3 detected in this study were closely related to those previously amplified in the north Pacific subtropical gyre (NPSG; Figure 5B).

In the core ODZ, the MGII archaea decreased drastically, accounting for <12% of the total archaeal 16S rRNA reads at the coastal station and <10% at the open ocean station and only at specific depths (Table 1 and Figure 3C, 4C), meaning that as for the Thaumarchaeota also the MGII were not well adapted to the core ODZ environment and in fact the group was only barely detected throughout the ODZ (Table 1 and Figure 3C, 4C).

In the deep oxic waters below the ODZ, the MGII archaea regained importance especially as MGII OTU-2 at the coastal station (29–32% of the total archaeal reads) and as MGII OTU-1 (14–21%) and MGII OTU-2 (6–13%) at the open ocean site (Table 1 and Figure 3C, 4C). The relative change in the composition of the MGII population from the shallow oxic waters and upper ODZ to the deep oxic waters was not as obvious as for the MGI population. Indeed, only the relative abundance of the MGII population was affected but not their composition (Table 1 and Figure 3C, 4C), indicating their ability to adapt to both the surface and to the deep sea rather than the differentiation of a specific deep MGII population. MGII-affiliated sequences have been detected in the deep waters of multiple locations worldwide; however, this group is typically more abundant in shallow waters (Frigaard et al., 2006; Baker et al., 2013; Deschamps et al., 2014). Interestingly, these studies the genomes of the MGII archaea thriving in deep waters did not harbor any homolog of the proteorhodopsin gene found in the shallow MGII ecotypes, but were found to hold multiple genes typical of heterotrophic prokaryotes involved in amino acid, carbohydrate and lipid transport and metabolism (Deschamps et al., 2014).

The appointment of specific IPLs to the MGII ETSP population was problematic because of the lack of pure cultures of this lineage and, accordingly, previous attempts to do so remain not conclusive to date (Turich et al., 2007; Schouten et al., 2008b, 2014; Lincoln et al., 2014). At both stations, MGII archaea and Thaumarchaeota occupied the same niche in the ETSP waters (Table 1 and Figure 3C, 4C). At the coastal station, copy numbers of MGII OTU-1 were positively correlated with those of *Ca*. Nitrosopelagicus, MGI OTU-1 and, especially *Nitrosopumilus*, whereas MGII OTU-2 correlated positively with the two “uncultured” MGI OTUs (Supplementary Figure S1A and Supplementary Table S6A). At the open ocean station, the MGII OTU-1 correlated highly positively with the two “uncultured” MGI OTUs. MGII OTU-2 scored highly positive with *Nitrosopumilus* and MGI OTU-1, and MGII OTU-3 with *Ca*. Nitrosopelagicus and *Nitrosopumilus* (Supplementary Figure S1B and Supplementary Table S6B). Because of the co-occurrence of the MGI and MGII groups in the ETSP waters, the positive correlation emerging from the archaea – IPL matrix between MGII OTU-1, MGII OTU-3 and some of the GDGT IPLs detected cannot be safely used to appoint these IPLs to the MGII affiliates (Figure 6 and Supplementary Table S5). Confirmation from pure culture experiments is needed as in the case of Thaumarchaeota.

### Marine Euryarchaeota Group III (MGIII) and DPANN Woesearchaeota Thrive in the ODZ of the ETSP Water Column

At both stations MGIII accounted for 40–70% of the total archaeal diversity in the core ODZ, depending on the depth (Table 1 and Figure 3C, 4C). MGIII archaea had been previously detected within the ETSP ODZ (Belmar et al., 2011; Stewart et al., 2011) and in suboxic and euxinic waters of the Black Sea (Sollai et al., 2018), although in these cases the group did not represent such a large part of the archaeal community as reported in this study (Table 1 and Figure 3C, 4C). Metagenomic studies have found evidence of fermentation-related genes, including those potentially involved in the metabolism of peptide and lipids, in the genomes of many MGIII archaea, suggesting that they might be facultative anaerobes (Martin-Cuadrado et al., 2008). Previous studies have also detected MGIII archaea in the marine photic zone; their genomes containing numerous photolyase and rhodopsin genes and suggesting a photoheterotrophic lifestyle (Martin-Cuadrado et al., 2008; Galand et al., 2009; Haro-Moreno et al., 2017). In the ETSP, the MGIII archaea were also present in the shallow oxic zone at both stations, with all three OTUs, but they represented only a minor group.

The three MGIII OTUs found in this study were closely related to sequences previously detected in the deep-sea waters and at various depths in the NESAP OMZ (Figure 5B). Therefore it seems that depth does not play a role in the distribution of these archaea, and oxygen concentration instead might have a key role. In the deep oxic waters of the ETSP, the MGIII archaea, mostly represented as MGIII OTU-3, became a minor group, accounting for not more than 15% of the total archaeal reads at both stations (Table 1 and Figure 3C, 4C). The presence of MGIII OTU-3 throughout the ETSP water column suggests higher adaptability of this OTU to different water conditions compared with the other MGIII OTUs detected.

Members of the DPANN superphylum are increasingly reported in anoxic water columns including the ETSP ODZ, and the Black Sea (Belmar et al., 2011; Sollai et al., 2018). In the ETSP, Belmar et al. (2011) detected a sequence related to the DPANN DHVE-5 group within the ODZ (Belmar et al., 2011). In this study, we report the presence of the DPANN Woesearchaeota DHVE-6 group as a major component of the archaeal ETSP ODZ community (accounting for 10–26% of the total archaeal reads in the core ODZ; Table 1 and Figure 3C, 4C). Recently, this same group was also found to be prominent in the euxinic waters of the Black Sea (Sollai et al., 2018), in the euxinic surface marine sediments of Lake Grevelingen in Netherlands (Lipsewers et al., 2018), in the anoxic surface sediments below the Arabian Sea ODZ (Besseling et al., 2018), as well as in lacustrine and estuarine systems (Ortiz-Alvarez and Casamayor, 2016; Lazar et al., 2017). Although their metabolic traits are still largely unknown, DPANN archaea are likely to depend greatly on others for their metabolic needs due to the reported small size of their cells and genomes (Rinke et al., 2013; Castelle et al., 2015).

At both stations the archaeol-IPLs were hardly detected in the shallow oxic waters and in the upper ODZ, but became the most prominent archaeal IPL group within the core ODZ (Tables 2, 3). This is the first study to report archaeol-IPLs within the core of one of the main ODZs worldwide. Archaeol-IPLs have been previously detected in suboxic and euxinic waters and in microbial mats in the Black Sea (Rossel et al., 2008; Sollai et al., 2018), but not in the ETNP (Xie et al., 2014), nor in the Arabian Sea ODZs (Schouten et al., 2012). In the ETSP, all archaeol-IPLs maximized within the core ODZ, while the GDGT-0–4 and crenarchaeol-IPLs instead drastically decreased (Tables 2, 3). The abundance of archaeol-IPLs correlated positively with each other in the IPL – IPL matrix but negatively with the GDGT-0–4 and crenarchaeol IPLs. This strongly suggests a common origin for the archaeol-IPLs distinct from that of the other IPLs (Supplementary Figure S2 and Supplementary Table S7). In addition, the copy numbers of both MGIII and DPANN archaea correlated positively with all archaeol-IPLs and negatively with the GDGT-0–4 and crenarchaeol-IPLs (Figure 6 and Supplementary Table S5). Since neither MGIII nor Woesearchaeota cultures are yet available, we suggest, by combining the result of the correlation analysis and the known metabolic features of the two archaeal groups, that MGIII archaea were primarily responsible for synthesizing the archaeol-IPLs detected in the ETSP ODZ. Most of the currently available genomes of DPANN archaea lack most, if not all, the genes coding for the enzymes of the archaeal lipid biosynthetic pathway with the exception of the genomes of the phylum *Ca*. Micrarchaeota and the genome of *Ca*. Iainarchaeum andersonii (Jahn et al., 2004; Villanueva et al., 2017). Therefore, it is likely that the DPANN Woesearchaeota (i.e., DHVE-6) detected in this study within the core of the ETSP ODZ did not have the ability to synthesize their own archaeal membrane lipids and were dependent on others to acquire a membrane, being MGIII the most likely candidate for the synthesis of the archaeol-IPLs. If so, the MGIII-derived lipids might also be the source of the membrane of the DPANN Woesearchaeota present in the ETSP ODZ. A recent study has described a relationship of dependency between an acidophilic archaeon of the ARMAN group belonging to the DPANN superphylum and a representative of Thermoplasmatales, the order to which the MGIII group belongs (Golyshina et al., 2017). According with this study many fundamental metabolic pathways, including phospholipid biosynthesis, were absent in the ARMAN archaeon and the authors assumed a mutualistic interaction between the two archaea that included gene transfer, an evidence that might corroborate our speculation of some sort of symbiosis/parasitic lifestyle between the MGIII and the DPANN Woesearchaeota.

## Conclusion

Our study expands the knowledge on the archaeal community thriving in the ETSP region, including its ODZ. We have found that this region harbors a highly diverse assemblage of archaeal lineages that are distributed across the water column according to a clear depth partitioning. This seems to be mostly depending on the oxygen changing concentration across the water column. We also shade light for the first time on the IPLs synthesized by these archaeal communities; the IPL distribution follows similar depth segregation as that of the archaeal lineages detected, and we tentatively assigned specific IPLs to specific archaeal groups based on statistical evidence. Specifically, HPH-crenarchaeol, the specific biomarker for living Thaumarchaeota, HPH-GDGT-0, DH-GDGT-3 and -4 were likely synthesized by Thaumarchaeota related to *Ca*. Nitrosopelagicus and *Nitrosopumilus* from the shallow oxic waters and the upper ODZ. MGII affiliated sequences were also abundant in the shallow oxic ETSP, but we could not assign specific IPLs to this group. MGIII dominated the archaeal community within the core ODZ together with DPANN archaea, but the former group was the one likely synthesizing the archaeol-IPLs there detected. MGIII might have also provided the membrane lipids to the DPANN Woesearchaeota group, which is predicted to lack lipid biosynthetic pathways. In the deep oxic waters below the ODZ, the composition of the deep MGI and MGII archaeal populations was different from the shallow one, being mostly represented by uncultured MGI and MGII OTUs. The MGI OTU-1 and MGI OTU-2 archaea synthesized a different suite of IPLs, which was characterized by higher proportions of HPH-GDGT-1, -2 and DH-GDGT-0 and crenarchaeol.

## Author Contributions

JSD, MS, and LV generated hypothesis and planned experiments. RK and his team performed the field work. MS and EH performed lipid analyses. LV performed the amplicon sequencing. MS, LV, and JSD interpreted the data and wrote the manuscript. All authors provided comments on the text.

## Conflict of Interest Statement

The authors declare that the research was conducted in the absence of any commercial or financial relationships that could be construed as a potential conflict of interest. The author MS declares her affiliation with Frontiers and the handling Editor states that the process nevertheless met the standards of a fair and objective review.

## Footnotes

1 http://www.arb-silva.de/

2 http://cran.r-project.org/
