# Supplementary material for: Archaeal Sources of Intact Membrane Lipid Biomarkers in the Oxygen Deficient Zone of the Eastern Tropical South Pacific
Source: Front Microbiol. 2019 Apr 11;10:765. doi: 10.3389/fmicb.2019.00765 (PMC6470261; doi:10.3389/fmicb.2019.00765)
Supplement: Supplementary file 1 [file Data_Sheet_1.pdf]

Supplementary Material

Archaeal sources of intact membrane lipid biomarkers in the oxygen deficient zone of the eastern tropical South Pacific

Martina Sollai, Laura Villanueva, Ellen C. Hopmans, Richard G. Keil and Jaap S. Sinninghe Damsté*

Correspondence: Corresponding Authors: jaap.damste@nioz.nl; laura.villanueva@nioz.nl

Table S1. The table lists the coastal (48, 51, 54, 58a, 58b, 61, 64a, 64b) and open ocean (11, 14, 15, 24) stations sampled for SPM using multiple *in situ* pump devices, during the NBP13-0 cruise (June–July 2013) aboard of the *R/V Nathaniel B. Palmer*. For each cast the depth (meters) of the sampling and the corresponding potential density (σ_θ, kg m^{-3}), the oxygen concentration measured (μmol kg^{-1}), the location (DMS, longitude and latitude), the date (dd/mm/yyyy) and the volume of water filtered by the pump deployed (Liters) are reported.

Station*	Depth of SPM sampling	Potential density	O_2	Location DMS (lon/lat)		Date	Amount of water filtered**
nr	(m)	(kg m^{-3})	(μmol kg^{-1})	S	W	dd/mm/yyyy	(L)
58b	22	25.63	211.8	20°44'59.7"	70°39'30.6"	19/07/2013	264
64a	60	26.05	21.5	20°42'23.2"	70°42'31.1"	21/07/2013	382
54	75	26.19	2.1	20°46'22.6"	70°48'51.6"	18/07/2013	452
64b	83	26.18	2.2	20°42'23.5"	70°42'29.0"	21/07/2013	1567
58a	100	26.30	2.2	20°45'23.3"	70°39'06.4"	19/07/2013	1834
51	135	26.30	2.2	20°28'27.6"	70°41'32.4"	17/07/2013	977
58a	150	26.40	2.2	20°45'23.3"	70°39'06.4"	19/07/2013	740
58b	175	26.45	2.2	20°44'59.7"	70°39'30.6"	19/07/2013	2286
64a	200	26.42	2.2	20°42'23.2"	70°42'31.1"	21/07/2013	1477
51	235	26.50	2.2	20°28'27.6"	70°41'32.4"	17/07/2013	3014
61	250	26.48	2.3	20°43'46.5	70°40'15.0"	20/07/2013	2668
61	300	26.61	2.4	20°43'46.5	70°40'15.0"	20/07/2013	745
64b	350	26.71	2.5	20°42'23.5"	70°42'29.0"	21/07/2013	549
48	500	26.95	14.6	20°33'07.1"	70°44'00.0"	16/07/2013	1031
48	1000	27.39	65.0	20°33'07.1"	70°44'00.0"	16/07/2013	4028
15	100	26.00	54.4	13°55'19.7"	81°16'38.8"	03/07/2013	2306
14	125	26.18	2.1	13°29'36.0"	81°12'06.2"	04/07/2013	3400
11	155	26.35	2.1	14°00'44.8"	81°07'10.1"	05/07/2013	851
14	175	26.35	2.1	13°29'36.0"	81°12'06.2"	06/07/2013	783
24	200	26.45	2.2	13°25'30.6"	81°13'50.5"	07/07/2013	3391
11	235	26.50	2.2	14°00'44.8"	81°07'10.1"	08/07/2013	3209
24	300	26.69	2.4	13°25'30.6"	81°13'50.5"	09/07/2013	875
24	500	26.99	15.9	13°25'30.6"	81°13'50.5"	10/07/2013	1036
24	1000	27.37	55.8	13°25'30.6"	81°13'50.5"	11/07/2013	4051

*Stations 58 and 64 were sampled twice

**Reading of the flow meter

Table S2. Physicochemical conditions in the ETSP water column at the coastal (st. 48, 54, 58, 61, 64) and open ocean (st. 14, 24, 26) stations, indicated as water depth (meters) and corresponding potential density (σ_θ, kg m^{-3}), oxygen concentration (O_2, µmol kg^{-1}), nitrate, (NO_3^-, µM), nitrite, (NO_2^-, µM) and ammonium, (NH_4^+, µM).

Station	Depth (m)	Estimated potential density (σ_θ) (kg m^{-3})	O_2 (µmol kg^{-1})	NO_3^- (µM)	NO_2^- (µM)	NH_4^+ (µM)
48	20	25.63	211.8	4.2	0.2	0.54
	94	26.27	2.1	11.9	3.8	0.06
	198	26.47	2.2	15.7	6.7	0.05
	230	26.50	2.3	17.1	6.5	0.03
	300	26.61	2.4	21.2	6.7	0.04
	500	26.95	14.7	41.2	0.0	0.06
54	30	25.85	123.1	11.4	0.5	1.48
	70	26.19	2.1	13.6	2.8	0.08
	115	26.31	2.1	9.7	5.6	0.03
	125	26.33	2.2	9.9	5.6	0.03
	281	26.50	2.3	16.4	6.6	0.05
	301	26.54	2.3	17.9	6.5	0.02
	323	26.60	2.4	21.0	5.6	0.02
	370	26.71	2.5	30.7	1.0	0.04
58	11	25.62	212	3.5	0.1	0.32
	19	25.61	214	4.3	0.2	0.35
	41	25.83	107	15.1	0.5	1.04
	51	25.96	44	19.5	0.6	0.70
	90	26.27	2	11.8	3.5	0.02
	100	26.30	2	10.2	5.3	0.03
	115	26.34	2	8.8	7.5	0.05
	200	26.42	2	13.5	7.2	0.12
	998	27.38	65	43.5	0.0	0.12
61	15	25.63	216	3.7	0.1	0.45
	29	25.69	192	6.9	0.2	0.83
	60	26.05	21	22.2	0.1	0.01
	79	26.19	2	15.2	1.6	0.02
	100	26.30	2	10.8	5.0	0.01
64	2	25.58	220	3.2	0.1	0.18
	15	25.61	217	3.6	0.1	0.36
	20	25.64	211	4.0	0.1	0.47
	25	25.66	201	5.5	0.2	0.61

	29	25.72	181	8.5	0.2	0.70
	40	25.97	43	21.8	0.3	0.09
	50	26.10	4	20.5	0.2	0.01
	60	26.17	2	15.9	1.6	0.02
	150	26.40	2	12.8	7.4	0.03
	239	26.46	2	15.6	7.2	0.02
	250	26.48	2	16.2	7.1	0.06
	325	26.59	2	20.8	6.6	0.01
	375	26.73	3	32.3	0.3	0.03
	2	25.37	214.1	2.6	0.20	0.20
	29	25.37	214.0	2.6	0.20	0.20
	35	25.37	213.3	2.6	0.20	0.20
	40	25.37	213.4	2.6	0.21	0.20
	64	25.46	191.7	5.5	0.23	0.26
	74	25.69	142.0	15.2	0.13	0.02
	159	26.37	2.3	19.6	0.60	0.00
14	250	26.55	2.3	20.0	6.73	0.01
	500	26.99	15.9	42.3	0.02	0.02
	699	27.19	23.4	45.7	0.02	0.04
	900	27.33	46.4	44.9	0.01	0.03
	900	27.33	46.4	44.8	0.02	0.04
	1000	27.39	55.7	44.1	0.01	0.02
	1300	27.53	76.2	42.3	0.02	0.02
	1401	27.56	81.1	41.9	0.02	0.06
	3	25.39	213.4	2.5	0.23	0.49
	20	25.38	212.8	2.5	0.22	0.50
	49	25.39	211.3	2.5	0.23	0.54
	79	25.92	93.2	20.7	0.06	0.06
	98	26.10	36.4	23.6	0.03	0.01
	124	26.25	5.5	19.9	0.03	0.06
	151	26.35	2.1	19.8	0.34	0.01
	201	26.49		23.0	1.68	0.03
	249	26.58		25.2	3.00	0.01
24	300	26.69		28.2	3.96	0.02
	350	26.77		35.7	0.04	0.02
	399	26.85		38.8	0.01	0.02
	500	27.01		43.0	0.01	0.03
	599	27.12		45.0	0.01	0.04
	800	27.28		45.7	0.01	0.04
	879	27.34		45.0	0.00	0.03
	1248	27.51		42.8	0.00	0.01
	1498	27.58		41.9	0.00	0.04
	1748	27.64		40.8	0.00	0.02

	2000	27.68		40.0	0.00	0.03
	2251	27.71		39.1	0.00	0.04
	2501	27.73		38.4	0.00	0.03
	2999	27.75		38.3	0.00	0.02
	3501	27.75		37.6	0.00	0.02
26	2	25.22	216.2	4.6	0.37	0.40
	51	25.26	212.4	2.5	0.22	0.38
	101	26.06	2.8	20.9	0.04	0.03
	124	26.18	2.1	9.4	8.84	0.01
	147	26.27	2.1	10.0	7.97	0.01
	175	26.35	2.1	14.7	4.33	0.02
	199	26.41	2.2	16.8	4.77	0.02
	226	26.47	2.2	20.1	5.44	0.01
	239	26.50	2.2	20.2	5.86	0.01
	252	26.53	2.2	21.6	5.91	0.02
	258	26.54	2.3	18.7	8.60	0.09
	270	26.56	2.3	18.8	8.81	0.02
	273	26.57	2.3	18.9	8.86	0.02
	280	26.59	2.3	20.6	7.99	0.02
	289	26.61	2.3	23.2	6.54	0.02
	301	26.62	2.4	24.4	6.01	0.02
	350	26.72	2.5	32.9	1.02	0.00
	398	26.82	3.8	37.9	0.02	0.05
	500	26.98	11.4	41.8	0.01	0.02
	600	27.09	19.9	44.1	0.01	0.04
	799	27.26	32.1	45.7	0.00	0.02
	1001	27.37	52.9	44.4	0.00	0.05
	1252	27.49	69.7	42.9	0.00	0.34
	1500	27.59	81.1	41.9	0.00	0.03

Table S3. Estimations of the archaeal 16S rRNA gene reads (copies L^{-1}) of specific archaeal groups detected in the ETSP SPM at (**a**) the coastal station and at the (**b**) open ocean station, obtained as described in the experimental procedures.

(**a**)

Depth (m)	MGI *Ca.*Nitrosopelagicus	MGI *Nitrosopumilus*	MGI uncultured OTU-1	MGI uncultured OTU-2	MGII uncultured OTU-1	MGII uncultured OTU-2	MGIII uncultured OTU-1	MGIII uncultured OTU-2	MGIII uncultured OTU-3	DPANN Woesearchaeota DHVE-6
22	8.4E+04	3.7E+04	2.8E+03	2.5E+03	1.0E+04	1.0E+04	0.0E+00	3.8E+03	9.4E+02	3.1E+02
*****60	0.0E+00	0.0E+00	0.0E+00	0.0E+00	0.0E+00	0.0E+00	0.0E+00	0.0E+00	0.0E+00	0.0E+00
75	4.2E+05	4.7E+05	6.0E+04	2.4E+04	1.0E+06	1.5E+05	6.0E+03	1.8E+05	6.6E+04	5.4E+04
83	1.0E+06	1.4E+06	1.4E+05	3.9E+04	6.3E+05	1.7E+05	2.6E+04	6.6E+04	1.2E+05	6.6E+04
100	7.0E+05	1.1E+06	6.8E+05	3.4E+05	4.2E+05	3.8E+05	1.9E+05	3.8E+05	1.0E+06	7.5E+05
135	1.9E+05	2.8E+05	3.8E+05	1.7E+05	3.4E+04	1.9E+05	3.6E+05	3.5E+05	1.1E+06	1.1E+06
150	3.4E+04	8.4E+04	1.3E+05	5.0E+04	0.0E+00	2.9E+05	2.2E+05	2.7E+05	8.0E+05	6.2E+05
175	2.7E+05	3.4E+05	9.9E+05	7.6E+05	5.7E+05	7.6E+05	1.4E+06	1.4E+06	3.0E+06	3.2E+06
200	2.4E+05	2.4E+05	1.8E+06	6.4E+05	0.0E+00	9.7E+05	2.7E+06	2.1E+06	4.6E+06	3.2E+06
235	1.5E+05	1.5E+05	1.5E+05	1.5E+05	0.0E+00	1.5E+05	1.5E+06	1.3E+06	3.2E+06	1.9E+06
250	0.0E+00	7.3E+05	2.3E+06	7.3E+05	1.2E+06	8.5E+05	3.3E+06	1.3E+06	5.0E+06	5.9E+06
300	0.0E+00	7.6E+04	9.1E+05	7.6E+05	7.6E+04	4.5E+05	2.3E+06	2.0E+06	4.9E+06	2.6E+06
350	4.1E+04	2.9E+05	6.2E+05	3.3E+05	0.0E+00	6.2E+05	2.7E+06	1.7E+06	3.4E+06	1.9E+06
500	1.4E+04	2.6E+05	1.3E+06	1.7E+06	1.9E+04	1.8E+06	7.2E+04	1.0E+05	2.6E+05	1.4E+04
1000	0.0E+00	2.0E+05	9.6E+05	9.2E+05	1.8E+04	1.0E+06	6.1E+04	6.1E+04	2.5E+05	1.8E+04

*****Samples from 60 m depth were not included into the Pearson correlation analysis

(**b**)

Depth (m)	MGI *Ca.*Nitrosopelagicus	MGI *Nitrosopumilus*	MGI uncultured OTU-1	MGI uncultured OTU-2	MGII uncultured OTU-1	MGII uncultured OTU-2	MGII uncultured OTU-3	MGIII uncultured OTU-1	MGIII uncultured OTU-2	MGIII uncultured OTU-3	DPANN Woesearchaeota DHVE-6
100	1.2E+06	5.8E+05	5.0E+05	7.8E+04	1.4E+05	6.6E+05	2.0E+05	0.0E+00	1.8E+05	6.1E+04	0.0E+00
125	4.2E+06	3.4E+06	3.6E+06	3.0E+05	6.8E+05	3.1E+06	1.0E+06	2.3E+04	1.4E+06	4.1E+05	6.8E+04
155	8.6E+05	2.4E+06	3.8E+06	6.2E+04	7.4E+05	2.6E+06	5.9E+05	1.2E+06	1.6E+06	2.1E+06	1.7E+06
175	1.9E+05	5.8E+05	2.2E+06	1.9E+05	1.9E+05	1.8E+06	0.0E+00	6.8E+06	5.4E+06	1.1E+07	1.1E+07
200	9.2E+05	3.2E+06	1.1E+07	1.1E+05	1.1E+06	5.3E+06	6.9E+05	1.0E+07	5.3E+06	1.6E+07	1.1E+07
235	1.1E+05	2.2E+05	8.7E+05	0.0E+00	1.1E+05	2.2E+05	0.0E+00	5.0E+06	3.0E+06	4.3E+06	3.0E+06
300	0.0E+00	9.7E+03	1.5E+05	9.7E+03	0.0E+00	9.7E+04	0.0E+00	9.0E+05	4.2E+05	6.5E+05	4.9E+05
500	1.3E+05	0.0E+00	1.4E+07	7.3E+05	3.7E+06	3.4E+06	3.5E+05	6.1E+05	7.7E+05	1.5E+06	2.9E+05
1000	2.9E+02	0.0E+00	7.2E+04	1.4E+04	3.5E+04	9.8E+03	8.6E+02	7.5E+03	1.7E+03	1.4E+04	2.6E+03

Table S4. Abundances of the archaeal IPLs (response units per Liter; r.u. L^{-1}) detected in the ETSP SPM at (**a**) the coastal station and at the (**b**) open ocean station.

(**a**)

Depth (m)	GDGT-0			GDGT-1			GDGT-2			GDGT-3	GDGT-4	Crenarchaeol			Archaeol			
	MH	DH	HPH	MH	DH	HPH	MH	DH	HPH	DH	DH	MH	DH	HPH	MH	DH	PG	PE
22	1.6E+04	0.0E+00	0.0E+00	0.0E+00	0.0E+00	0.0E+00	0.0E+00	1.3E+05	0.0E+00	0.0E+00	0.0E+00	4.4E+04	0.0E+00	0.0E+00	0.0E+00	0.0E+00	0.0E+00	0.0E+00
*****60	1.7E+06	8.3E+06	1.0E+08	2.0E+05	2.3E+07	5.9E+06	6.1E+04	4.4E+07	1.2E+06	9.8E+06	2.4E+06	2.4E+06	1.0E+07	6.7E+07	0.0E+00	0.0E+00	4.3E+06	1.6E+06
75	3.7E+06	9.1E+06	3.1E+08	4.4E+05	3.0E+07	2.7E+07	1.8E+05	4.9E+07	6.5E+06	1.2E+07	5.6E+06	3.5E+06	6.7E+06	2.5E+08	0.0E+00	0.0E+00	2.4E+07	2.6E+06
83	3.1E+06	1.0E+07	3.6E+08	4.9E+05	3.5E+07	3.7E+07	1.4E+05	5.7E+07	6.9E+06	1.2E+07	3.5E+06	5.0E+06	1.0E+07	2.9E+08	0.0E+00	0.0E+00	1.9E+07	2.8E+06
100	5.2E+05	2.8E+06	9.2E+07	6.8E+04	9.4E+06	7.9E+06	2.5E+04	1.6E+07	1.1E+06	1.6E+06	7.1E+05	1.1E+06	3.3E+06	6.5E+07	2.4E+04	8.4E+05	2.9E+07	5.7E+06
135	4.0E+05	2.2E+06	8.5E+07	8.0E+04	5.1E+06	1.2E+07	1.2E+04	1.4E+07	2.8E+06	5.7E+05	2.6E+05	1.1E+06	3.6E+06	7.7E+07	1.5E+05	2.2E+06	7.5E+07	1.2E+07
150	1.8E+05	2.7E+05	8.0E+06	4.9E+04	1.1E+06	0.0E+00	1.8E+04	3.6E+06	0.0E+00	0.0E+00	0.0E+00	6.3E+05	0.0E+00	8.0E+06	1.6E+05	6.5E+05	3.0E+07	7.3E+06
175	3.5E+05	0.0E+00	1.5E+07	7.7E+04	3.1E+06	1.4E+06	1.6E+04	6.2E+06	0.0E+00	0.0E+00	0.0E+00	9.3E+05	1.8E+06	1.1E+07	2.6E+05	1.2E+06	4.1E+07	1.1E+07
200	1.3E+05	7.0E+05	7.6E+06	4.1E+04	2.3E+06	0.0E+00	1.1E+04	7.5E+06	0.0E+00	0.0E+00	0.0E+00	6.9E+05	1.7E+06	8.4E+06	3.4E+05	2.4E+06	6.8E+07	1.7E+07
235	1.4E+05	0.0E+00	1.4E+07	3.6E+04	1.3E+06	1.3E+06	1.5E+04	4.4E+06	0.0E+00	0.0E+00	0.0E+00	3.9E+05	1.2E+06	9.9E+06	2.3E+05	1.0E+06	4.7E+07	8.0E+06
250	1.3E+05	0.0E+00	5.9E+06	2.3E+04	6.7E+05	2.2E+05	5.1E+03	3.0E+06	0.0E+00	0.0E+00	0.0E+00	3.7E+05	8.3E+05	5.8E+06	2.2E+05	1.5E+06	4.0E+07	1.0E+07
300	7.7E+04	6.9E+04	2.5E+07	2.0E+04	8.2E+05	2.8E+06	3.9E+03	3.9E+06	0.0E+00	0.0E+00	4.4E+04	3.5E+05	5.4E+05	2.1E+07	2.9E+05	1.3E+06	6.6E+07	1.1E+07
350	1.1E+05	0.0E+00	1.1E+07	3.3E+04	1.1E+06	1.0E+06	8.2E+03	3.8E+06	0.0E+00	0.0E+00	0.0E+00	4.5E+05	7.3E+05	9.2E+06	3.1E+05	1.5E+06	7.4E+07	8.6E+06
500	1.6E+05	3.1E+06	9.6E+07	3.8E+04	8.0E+06	2.1E+07	1.3E+04	2.0E+07	5.4E+06	1.7E+05	2.5E+05	3.9E+05	1.1E+07	7.3E+07	2.3E+03	0.0E+00	3.4E+06	6.7E+05
1000	6.3E+05	4.2E+06	4.3E+07	1.5E+05	1.0E+07	5.7E+06	1.7E+04	2.7E+07	0.0E+00	1.2E+05	5.5E+05	7.6E+05	6.9E+06	1.8E+07	1.9E+03	0.0E+00	5.7E+06	

*****Samples from 60 m depth were not included into the Pearson correlation analysis

(**b**)

Depth (m)	GDGT-0			GDGT-1			GDGT-2			GDGT-3	GDGT-4	Crenarchaeol			Archaeol			
	MH	DH	HPH	MH	DH	HPH	MH	DH	HPH	DH	DH	MH	DH	HPH	MH	DH	PG	PE
100	8.3E+05	1.6E+06	4.5E+07	1.2E+05	1.0E+07	3.2E+06	5.4E+04	2.5E+07	0.0E+00	2.6E+06	5.0E+05	1.7E+06	9.8E+06	3.1E+07	0.0E+00	0.0E+00	3.1E+06	1.7E+06
125	6.5E+05	2.5E+06	1.3E+08	1.2E+05	8.2E+06	1.5E+07	5.5E+04	2.0E+07	2.0E+06	2.5E+06	5.4E+05	1.0E+06	7.5E+06	1.1E+08	0.0E+00	0.0E+00	3.6E+06	8.7E+05
155	2.9E+05	8.7E+05	2.0E+07	5.2E+04	3.4E+06	1.9E+06	2.5E+04	8.3E+06	1.2E+05	5.3E+05	2.0E+05	5.8E+05	2.3E+06	1.6E+07	3.4E+04	6.8E+04	7.0E+06	1.3E+06
175	2.9E+05	6.7E+05	1.7E+06	3.3E+04	1.1E+06	0.0E+00	1.7E+04	5.5E+06	0.0E+00	0.0E+00	0.0E+00	5.3E+05	2.2E+06	9.0E+05	1.6E+05	6.4E+05	3.4E+07	4.2E+06
200	1.8E+05	8.3E+05	2.3E+07	3.3E+04	1.8E+06	3.5E+06	2.3E+04	8.5E+06	3.1E+05	3.7E+05	1.6E+05	5.9E+05	2.5E+06	2.6E+07	1.8E+05	4.7E+05	4.3E+07	3.6E+06
235	5.9E+04	1.2E+05	6.2E+06	9.9E+03	1.1E+06	0.0E+00	6.0E+03	3.1E+06	0.0E+00	5.8E+04	0.0E+00	2.4E+05	6.6E+05	5.6E+06	1.5E+05	4.3E+05	2.9E+07	5.6E+06
300	1.9E+05	8.2E+05	8.0E+06	4.4E+04	2.0E+06	8.7E+05	2.4E+04	7.1E+06	6.6E+04	0.0E+00	0.0E+00	5.2E+05	2.2E+06	9.3E+06	2.1E+05	8.4E+05	4.5E+07	4.0E+06
500	1.2E+05	3.0E+06	8.5E+07	2.5E+04	8.0E+06	1.8E+07	3.7E+03	2.7E+07	5.1E+06	2.7E+05	3.0E+05	2.1E+05	1.2E+07	5.7E+07	0.0E+00	0.0E+00	2.7E+06	9.2E+05
1000	3.4E+05	2.7E+06	2.4E+07	9.6E+04	8.2E+06	4.0E+06	4.8E+04	2.2E+07	4.0E+05	2.0E+05	0.0E+00	4.5E+05	5.1E+06	9.4E+06	0.0E+00	0.0E+00	8.7E+06	1.2E+06

Table S5. Values of the Pearson correlation coefficients (r values) obtained from the correlation matrix created using as variables the total archaeal 16S rRNA gene reads of the archaeal groups (copies L^{-1}) and the absolute abundance of the archaeal IPLs (response units per Liter; r.u. L^{-1}) detected in the ETSP at the (**a**) coastal and (**b**) open ocean stations.

(**a**)

	GDGT-0			GDGT-1			GDGT-2			GDGT-3	GDGT-4	Crenarchaeol			Archaeol			
	MH	DH	HPH	MH	DH	HPH	MH	DH	HPH	DH	DH	MH	DH	HPH	MH	DH	PG	PE
MGI *Ca.*Nitrosopelagicus	0.68	0.70	0.77	0.72	0.77	0.67	0.67	0.70	0.58	0.74	0.61	0.83	0.46	0.76	-0.38	-0.22	-0.15	-0.16
MGI *Nitrosopumilus*	0.55	0.60	0.65	0.59	0.66	0.59	0.52	0.61	0.50	0.61	0.48	0.69	0.45	0.63	-0.34	-0.16	-0.16	-0.15
MGI uncultured OTU-1	-0.39	-0.33	-0.38	-0.38	-0.35	-0.30	-0.40	-0.30	-0.28	-0.40	-0.39	-0.37	-0.06	-0.39	0.41	0.43	0.19	0.46
MGI uncultured OTU-2	-0.37	-0.18	-0.27	-0.34	-0.24	-0.05	-0.38	-0.14	-0.02	-0.40	-0.35	-0.38	0.34	-0.29	0.06	-0.01	-0.15	0.02
MGII uncultured OTU-1	0.56	0.42	0.48	0.50	0.48	0.35	0.56	0.41	0.36	0.55	0.57	0.49	0.17	0.48	-0.16	-0.13	-0.15	-0.06
MGII uncultured OTU-2	-0.33	-0.14	-0.25	-0.29	-0.20	-0.04	-0.33	-0.09	0.00	-0.36	-0.31	-0.33	0.36	-0.27	0.07	0.02	-0.15	0.04
MGIII uncultured OTU-1	-0.42	-0.56	-0.49	-0.43	-0.50	-0.54	-0.40	-0.53	-0.53	-0.40	-0.42	-0.39	-0.56	-0.47	0.87	0.71	0.68	0.74
MGIII uncultured OTU-2	-0.42	-0.57	-0.48	-0.43	-0.50	-0.55	-0.39	-0.53	-0.54	-0.40	-0.41	-0.38	-0.57	-0.46	0.94	0.73	0.77	0.81
MGIII uncultured OTU-3	-0.46	-0.61	-0.52	-0.48	-0.54	-0.58	-0.44	-0.57	-0.57	-0.44	-0.46	-0.42	-0.59	-0.50	0.91	0.73	0.71	0.80
DPANN Woesearchaeota	-0.39	-0.54	-0.46	-0.41	-0.48	-0.52	-0.38	-0.51	-0.50	-0.38	-0.40	-0.35	-0.53	-0.44	0.75	0.67	0.54	0.72

(**b**)

	GDGT-0			GDGT-1			GDGT-2			GDGT-3	GDGT-4	Crenarchaeol			Archaeol			
	MH	DH	HPH	MH	DH	HPH	MH	DH	HPH	DH	DH	MH	DH	HPH	MH	DH	PG	PE
MGI *Ca.*Nitrosopelagicus	0.64	0.30	0.80	0.64	0.39	0.49	0.61	0.26	0.14	0.80	0.77	0.52	0.29	0.87	-0.39	-0.40	-0.39	-0.43
MGI *Nitrosopumilus*	0.26	-0.04	0.41	0.24	-0.05	0.20	0.30	-0.13	-0.06	0.41	0.46	0.25	-0.11	0.53	-0.06	-0.19	-0.04	-0.24
MGI uncultured OTU-1	-0.35	0.30	0.35	-0.38	0.03	0.61	-0.44	0.24	0.71	-0.14	0.23	-0.31	0.39	0.34	-0.08	-0.18	-0.05	-0.24
MGI uncultured OTU-2	-0.10	0.61	0.64	-0.13	0.36	0.87	-0.30	0.54	0.96	0.07	0.41	-0.20	0.73	0.56	-0.39	-0.36	-0.40	-0.44
MGII uncultured OTU-1	-0.30	0.51	0.47	-0.30	0.25	0.77	-0.45	0.46	0.92	-0.10	0.30	-0.33	0.62	0.40	-0.32	-0.35	-0.32	-0.40
MGII uncultured OTU-2	-0.10	0.13	0.39	-0.16	-0.07	0.46	-0.17	0.05	0.41	0.08	0.36	-0.07	0.17	0.45	-0.02	-0.15	0.01	-0.24
MGII uncultured OTU-3	0.31	0.28	0.71	0.32	0.23	0.56	0.31	0.19	0.31	0.53	0.68	0.25	0.24	0.79	-0.33	-0.42	-0.31	-0.49
MGIII uncultured OTU-1	-0.45	-0.60	-0.45	-0.59	-0.71	-0.39	-0.46	-0.63	-0.30	-0.42	-0.40	-0.29	-0.55	-0.35	0.71	0.55	0.71	0.65
MGIII uncultured OTU-2	-0.36	-0.58	-0.34	-0.53	-0.71	-0.32	-0.44	-0.65	-0.26	-0.34	-0.32	-0.26	-0.53	-0.23	0.63	0.50	0.60	0.59
MGIII uncultured OTU-3	-0.37	-0.50	-0.38	-0.51	-0.64	-0.31	-0.39	-0.54	-0.24	-0.37	-0.32	-0.23	-0.46	-0.28	0.63	0.49	0.65	0.52
DPANN Woesearchaeota	-0.33	-0.53	-0.43	-0.49	-0.66	-0.38	-0.37	-0.57	-0.30	-0.37	-0.36	-0.21	-0.50	-0.33	0.64	0.53	0.66	0.54

Table S6. Values of the Pearson correlation coefficients (r values) obtained from the correlation matrix created using as variables the total archaeal 16S rRNA gene reads of the archaeal groups (copies L^{-1}) detected in the ETSP at the (**a**) coastal and (**b**) open ocean stations.

(**a**)

	Thaumarchaeota Marine Group I				Euryarchaeota Marine Group II		Euryarchaeota Marine Group III			DPANN
	*Ca.*Nitrosopelagicus	*Nitrosopumilus*	uncultured OTU-1	uncultured OTU-2	uncultured OTU-1	uncultured OTU-2	uncultured OTU-1	uncultured OTU-2	uncultured OTU-3	Woesearchaeota DHVE-6
MGI *Ca.*Nitrosopelagicus										
MGI *Nitrosopumilus*	0.86									
MGI uncultured OTU-1	-0.32	0.05								
MGI uncultured OTU-2	-0.40	-0.17	0.69							
MGII uncultured OTU-1	0.38	0.61	0.25	-0.10						
MGII uncultured OTU-2	-0.37	-0.12	0.73	0.95	-0.09					
MGIII uncultured OTU-1	-0.36	-0.13	0.66	0.19	0.12	0.21				
MGIII uncultured OTU-2	-0.30	-0.24	0.49	0.16	-0.07	0.14	0.92			
MGIII uncultured OTU-3	-0.34	-0.17	0.63	0.20	0.08	0.16	0.96	0.96		
DPANN Woesearchaeota	-0.29	0.00	0.74	0.20	0.38	0.19	0.89	0.76	0.90	

(**b**)

	Thaumarchaeota Marine Group I				Euryarchaeota Marine Group II			Euryarchaeota Marine Group III			DPANN
	*Ca.*Nitrosopelagicus	*Nitrosopumilus*	uncultured OTU-1	uncultured OTU-2	uncultured OTU-1	uncultured OTU-2	uncultured OTU-3	uncultured OTU-1	uncultured OTU-2	uncultured OTU-3	Woesearchaeota DHVE-6
MGI *Ca.*Nitrosopelagicus											
MGI *Nitrosopumilus*	0.74										
MGI uncultured OTU-1	0.01	0.30									
MGI uncultured OTU-2	0.14	-0.02	0.75								
MGII uncultured OTU-1	-0.04	0.02	0.90	0.92							
MGII uncultured OTU-2	0.37	0.73	0.85	0.50	0.60						
MGII uncultured OTU-3	0.82	0.91	0.47	0.31	0.32	0.77					
MGIII uncultured OTU-1	-0.21	0.29	0.33	-0.16	-0.05	0.47	0.02				
MGIII uncultured OTU-2	-0.06	0.36	0.28	-0.07	-0.05	0.49	0.10	0.94			
MGIII uncultured OTU-3	-0.16	0.35	0.40	-0.06	0.02	0.57	0.09	0.98	0.95		
DPANN Woesearchaeota	-0.18	0.30	0.30	-0.10	-0.06	0.49	0.02	0.95	0.96	0.99	

Table S7. Pearson correlation coefficients (r values) obtained from the correlation matrix created using as variables the absolute abundance of the archaeal IPLs (response units per Liter; r.u. L^{-1}) detected in the ETSP at the (**a**) coastal and (**b**) open ocean stations.

(**a**)

		GDGT-0			GDGT-1			GDGT-2			GDGT-3	GDGT-3	Crenarchaeol			Archaeol			
		MH	DH	HPH	MH	DH	HPH	MH	DH	HPH	DH	DH	MH	DH	HPH	MH	DH	PG	PE
GDGT-0	MH																		
	DH	0.93																	
	HPH	0.95	0.96																
GDGT-1	MH	0.98	0.95	0.95															
	DH	0.96	0.99	0.98	0.98														
	HPH	0.83	0.93	0.95	0.85	0.92													
GDGT-2	MH	0.99	0.91	0.94	0.96	0.94	0.82												
	DH	0.93	1.00	0.96	0.96	0.99	0.93	0.90											
	HPH	0.80	0.87	0.91	0.79	0.86	0.98	0.80	0.86										
GDGT-3	DH	0.99	0.92	0.97	0.97	0.96	0.86	0.98	0.91	0.82									
GDGT-4	DH	0.99	0.91	0.92	0.94	0.92	0.80	0.99	0.89	0.80	0.97								
Crenarchaeol	MH	0.94	0.92	0.96	0.97	0.96	0.86	0.92	0.92	0.80	0.96	0.88							
	DH	0.60	0.81	0.74	0.66	0.76	0.88	0.57	0.83	0.84	0.59	0.57	0.62						
	HPH	0.95	0.95	1.00	0.95	0.97	0.95	0.94	0.94	0.92	0.97	0.93	0.96	0.71					
Archaeol	MH	-0.50	-0.67	-0.57	-0.50	-0.60	-0.63	-0.47	-0.63	-0.60	-0.49	-0.51	-0.43	-0.67	-0.54				
	DH	-0.46	-0.54	-0.47	-0.47	-0.52	-0.49	-0.46	-0.53	-0.46	-0.45	-0.47	-0.36	-0.55	-0.43	0.82			
	PG	-0.27	-0.40	-0.29	-0.29	-0.36	-0.35	-0.26	-0.39	-0.34	-0.26	-0.28	-0.19	-0.50	-0.25	0.85	0.91		
	PE	-0.38	-0.51	-0.41	-0.39	-0.46	-0.46	-0.36	-0.48	-0.43	-0.37	-0.40	-0.28	-0.53	-0.38	0.88	0.95	0.87	

(**b**)

		GDGT-0			GDGT-1			GDGT-2			GDGT-3	GDGT-3	Crenarchaeol			Archaeol			
		MH	DH	HPH	MH	DH	HPH	MH	DH	HPH	DH	DH	MH	DH	HPH	MH	DH	PG	PE
GDGT-0	MH																		
	DH	0.33																	
	HPH	0.47	0.73																
GDGT-1	MH	0.93	0.52	0.53															
	DH	0.70	0.84	0.67	0.78														
	HPH	0.15	0.81	0.91	0.24	0.61													
GDGT-2	MH	0.88	0.40	0.39	0.97	0.66	0.08												
	DH	0.53	0.91	0.66	0.62	0.96	0.71	0.48											
	HPH	-0.14	0.71	0.69	-0.08	0.44	0.92	-0.26	0.61										
GDGT-3	DH	0.93	0.34	0.67	0.84	0.69	0.36	0.77	0.54	0.05									
GDGT-4	DH	0.76	0.48	0.82	0.66	0.72	0.63	0.54	0.65	0.39	0.91								
Crenarchaeol	MH	0.94	0.10	0.29	0.80	0.56	-0.01	0.77	0.40	-0.26	0.89	0.72							
	DH	0.47	0.81	0.72	0.47	0.87	0.80	0.29	0.94	0.75	0.55	0.73	0.38						
	HPH	0.46	0.61	0.98	0.51	0.56	0.85	0.40	0.54	0.60	0.68	0.81	0.31	0.61					
Archaeol	MH	-0.56	-0.79	-0.65	-0.66	-0.89	-0.61	-0.52	-0.84	-0.47	-0.58	-0.66	-0.37	-0.75	-0.53				
	DH	-0.50	-0.71	-0.62	-0.58	-0.83	-0.58	-0.46	-0.78	-0.44	-0.56	-0.66	-0.33	-0.69	-0.52	0.97			
	PG	-0.54	-0.71	-0.63	-0.59	-0.84	-0.59	-0.44	-0.78	-0.47	-0.58	-0.67	-0.35	-0.73	-0.52	0.98	0.95		
	PE	-0.52	-0.84	-0.67	-0.65	-0.83	-0.66	-0.54	-0.82	-0.51	-0.51	-0.65	-0.33	-0.73	-0.58	0.90	0.84	0.84	

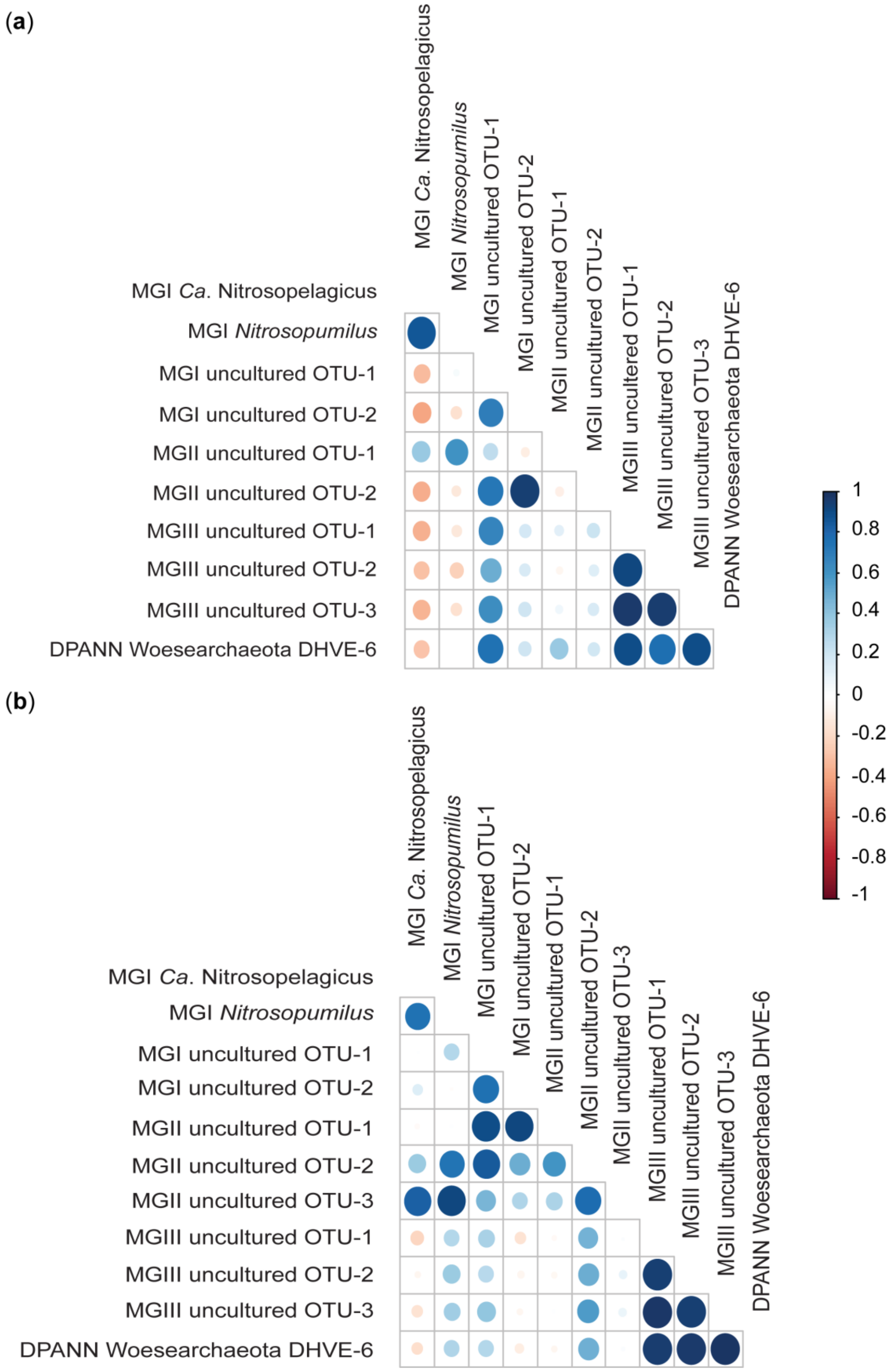

Figure S1. Dot plot of the correlation matrix obtained by applying a Pearson analysis to the total archaeal 16S rRNA gene reads (copies L^{-1}) of the archaeal groups detected in the ETSP at the (**a**) coastal and (**b**) open ocean stations. Dark blue corresponds to +1 r values, indicating a strong positive linear correlation between archaeal groups; white corresponds to 0 r values, indicating that no correlation exists; dark red corresponds to -1 r values, indicating a strong negative linear correlation.

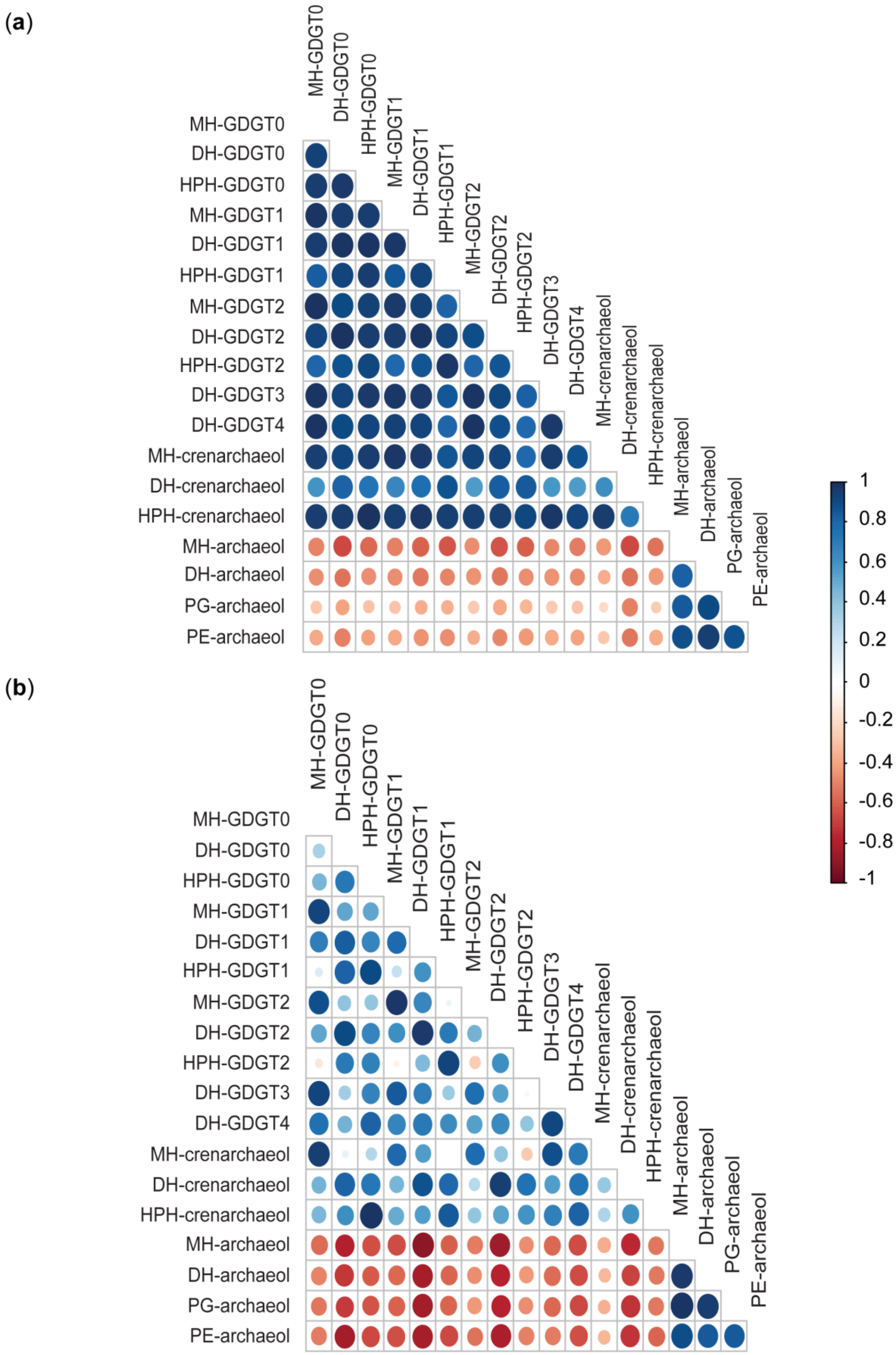

Figure S2. Dot plot of the correlation matrix obtained by applying a Pearson analysis to the archaeal IPLs (response units per Liter; r.u. L^{-1}) detected in the ETSP, at the (**a**) coastal and (**b**) open ocean stations. Dark blue corresponds to +1 r values, indicating a strong positive linear correlation between archaeal groups; white corresponds to 0 r values, indicating that no correlation exists; dark red corresponds to -1 r values, indicating a strong negative linear correlation.
